# Network pharmacology to dissect the mechanisms of Yinlai Decoction for pneumonia

**DOI:** 10.1186/s12906-020-02954-z

**Published:** 2020-06-03

**Authors:** Jingnan Xu, Chen Bai, Ling Huang, Tiegang Liu, Yuxiang Wan, Zian Zheng, Xueyan Ma, Fei Gao, He Yu, Xiaohong Gu

**Affiliations:** 1 School of Traditional Chinese Medicine, Beijing University of Chinese Medicine, Beijing, 100029 People’s Republic of China; 2 Department of Acupuncture and Mini-invasive Oncology, Beijing University of Chinese Medicine Third Affiliated Hospital, Beijing, 100029 People’s Republic of China; 3 School of Basic Medical Sciences, Guiyang University of Chinese Medicine, Guiyang, Guizhou 550025 People’s Republic of China

## Abstract

**Background:**

Pneumonia is a common respiratory disorder, which brings an enormous financial burden to the medical system. However, the current treatment options for pneumonia are limited because of drug resistance and side effects. Our previous study preliminarily confirmed that Yinlai Decoction (YD), a common prescription for pneumonia in clinical practice, can regulate the expression of inflammatory factors, but the mechanisms are unknown yet.

**Methods:**

In our work, a method named network pharmacology was applied, which investigated the underlying mechanisms of herbs based on a variety of databases. We obtained bioactive ingredients of YD on TCMSP database and collected potential targets of these ingredients by target fishing. Then the pneumonia-related targets database was built by TTD, Drugbank, HPO, OMIM, and CTD. Based on the matching targets between YD and pneumonia, the PPI network was built by STRING to analyze the interactions among these targets and then input into Cytoscape for further topological analysis. DAVID and KEGG were utilized for GO and pathway enrichment analysis. Then rat model based on LPS stimulated pneumonia was used to verify the possible mechanism of YD in treating pneumonia.

**Results:**

Sixty-eight active ingredients, 103 potential targets and 8 related pathways, which likely exert a number of effects, were identified. Three networks were constructed using Cytoscape, which were herb-component-network, YD-pneumonia target network, and herb-component-YD target-pneumonia network. YD was verified to treat LPS-induced pneumonia by regulating the inflammatory factor IL-6, which was a predicted target.

**Conclusion:**

Network analysis indicated that YD could alleviate the symptoms and signs of pneumonia through regulating host immune inflammatory response, angiogenesis and vascular permeability, the barrier function of the airway epithelial cells, hormone releasing and cell growth, proliferation, and apoptosis.

## Background

Globally, pneumonia is one of the leading causes of morbidity and mortality and accounted for 3 million deaths worldwide in 2016 [1], and imparts an enormous financial burden to the medical system [2]. The coronavirus disease 2019 (COVID-19) pneumonia has been declared a pandemic by the World Health Organization (WHO) on March 11, 2020 [3]. It has triggered enormous human casualties and serious economic loss and attracted the world’s enormous attention to pneumonia. Pneumonia is a syndrome with common clinical symptoms such as fever, cough, dyspnea, and fixed moist rale in the lungs, primarily caused by host immunoreaction to respiratory pathogens including bacteria, virus, mycoplasma, and chlamydia, etc. Currently, the main treatments for pneumonia include antibacterial, antivirus, immune inhibition, analgesics, antipyretics, and antihistamine. However, due to drug resistance and the limited efficacy of specific symptoms, their clinical application is limited [4].

Traditional Chinese medicine (TCM) holds a holistic and integrative perspective on health maintenance. Therapeutic effect of Chinese herbal represents the outcome achieved from the combined effects of multi-targets and multi-pathways, which help to reach a state of individual internal physiological harmony. Yinlai Decoction (YD) comprises seven herbs: *Lonicera japonica Thunb* (*L. japonica Thunb*, JYH), *Raphanus sativus L* (*R. sativus L*, LFZ), *Forsythia suspensa (Thunb.) Vahl* (*F. suspensa*, LQ), *Scutellaria baicalensis Georgi* (*S. baicalensis Georgi*, HQ), *Peucedanum praeruptorum Dunn* (*P. praeruptorum Dunn*, QH), *Houttuynia cordata Thunb* (*H. cordata Thunb*, YXC), *Trichosanthes rosthornii Harms* (*F. rosthornii Harms*, GL), and is commonly used for respiratory tract infection in clinical practice, such as influenza and pneumonia. Our previous study preliminarily confirms that YD can alleviate the symptoms of influenza virus infection and regulate the level of inflammatory factors such as interleukin (IL)-10 and tumor necrosis factor (TNF)-α [5]. However, the effective components and the mechanisms of YD in the treatment of pneumonia were still unclear and further exploration is necessary.

Network pharmacology is characterized by discovering TCM from a systematic perspective and at the molecular level. A growing evidence shows that the network pharmacology strategy can be a powerful approach to modern research on TCM [6, 7]. Based on the interaction network analysis of “disease-target-drug”, the intervention and influence of drugs on disease are systematically and comprehensively observed [8]. Comparing with the classic trial and error method, network pharmacology can provide a system-level of understanding the interaction mechanism between drugs and complex diseases in a high-throughput manner and within less time. Different from the current“one target, one drug” paradigm, the mechanisms of “multi-targets and multi-components” on the disease can be revealed based on this approach [5]. In brief, network pharmacology is an attractive modality that assists in drug development [9].

In this work, active ingredients prediction, target fishing, and network construction were carried out to evaluate active ingredients and targets of YD, so as to reveal the mechanisms of YD for pneumonia and provide a novel method for clinical practice.

## Methods

**Fig. 1:**
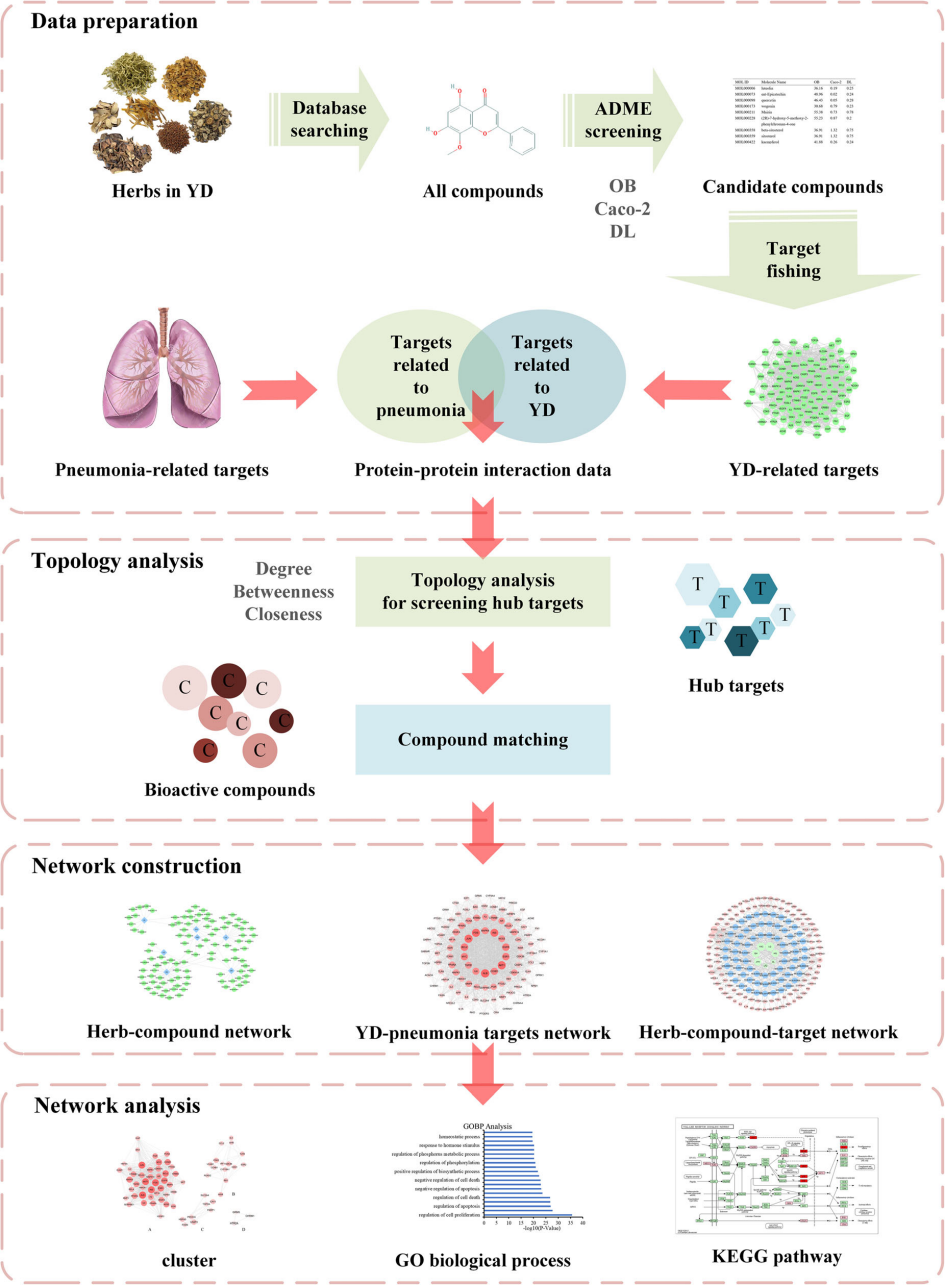
Flow chart of pharmacological analysis of YD for pneumonia treatment

The basic process of network pharmacology includes: identification of ingredients in the compound, acquisition of disease-related targets, construction of compound-ingredient-target-disease network, bioinformatics annotation and experimental verification. The visible procedure of analysis for the mechanism of YD for pneumonia based on network pharmacology was as follows (as displayed in Fig. 1).

### Data preparation

Totally, 625 chemical ingredients of 7 herbs in YD were manually extracted from Traditional Chinese Medicine Systems Pharmacology Database (TCMSP, http://lsp.nwu.edu.cn/tcmsp.php, Version 2.3). TCMSP have collected all the herbs registered in Chinese pharmacopoeia (2010) based on critically examined pharmacology and clinical knowledge, and is a pharmacological platform of TCM which covered a variety of pharmacokinetic parameters, targets, and related diseases [10].

#### Bioactive ingredients prediction

ADME (absorption, distribution, metabolism, and excretion) evaluation system can save the time and cost of the experiments by screening the active ingredients employing the above stated databases. Thus, three pharmacokinetic parameters including oral bioavailability (OB), Caco-2 cell permeability (Caco-2) and drug-likeness (DL) were selected to identify the active ingredients of YD.

OB refers to oral bioavailability, which is defined as the rate of active ingredient being absorbed from a drug product and becoming available at the site of action [11]. Caco-2 permeability is widely used to predict the intestinal ability to absorb components [12]. DL is a qualitative concept used in drug design and applied to assess the balance among molecular properties, which can explain how “druglike” a compound is [13]. In this study, OB ≥ 30%, Caco-2 > − 0.4 and DL ≥ 0.18 were the screening criteria for our ADME evaluation system. The components qualified for these criteria were regarded as the candidate ingredients for follow-up analysis.

Thirty-six molecules were obtained from *S. baicalensis Georgi*, 23 from *L. japonica Thunb*, 21 from *F. suspensa*, 18 from *P. praeruptorum Dunn*, 11 from *F. rosthornii Harms*, 6 from *H. cordata Thunb*, and 3 from *R. sativus L*. Many overlapping molecules among the seven herbs were removed.

#### Target fishing

We input all the bioactive components into TCMSP and obtained the targets of each component. Several components had no relevant targets, such as (MOL000358) beta-sitosterol, (MOL000359) sitosterol, (MOL000449) Stigmasterol, (MOL002926) dihydrooroxylin A, (MOL003365) Lactucasterol, (MOL003281) 20(S)-dammar-24-ene-3β,20-diol-3-acetate, (MOL003975) icosa-11,14,17-trienoic acid methyl ester and (MOL013101) rutarin_qt, which were drawn in ChemBioDraw version 16, saved as “mol2” file and imported into ChemMapper (http://lilab.ecust.edu.cn/chemmapper/, entering at Jan 2018). It is a website that discovers drug through computer methods in view of the notion that ingredients with similar structures may have similar targeting properties [14]. The Universal Protein Resource Knowledge base (UniProt Knowledge base, http://www.uniprot.org/, entering at Jan 2018) is applied to unify the standard nomenclature, which is a collection of information of functional proteins with accurate, consistent and rich annotation [15]. Filtered by “swiss-prot reviewed” and “*Homo sapiens*”, the official symbol of these proteins was acquired. By far, we acquired the bioactive components and potential targets of YD.

#### Pneumonia targets

Genes related to pneumonia were acquired from the following existing databases. The former two databases were used to collect relevant targets for drugs currently on the market or under research to treat pneumonia. The latter three databases were used to collect as comprehensive a collection of pneumonia-related targets as possible from authoritative databases. (1) Therapeutic Target Database (TTD, https://db.idrblab.org/ttd/, version Sep 15th, 2017) - a database for collecting information on 2544 approved drugs, 8103 drugs in clinical trials and 18,923 drugs under investigation [16]. (2) Drugbank (https://www.drugbank.ca/, version 5.1.1) - a database for collecting information on 4100 approved or experimental drug products [17]. (3) Human Phenotype Ontology (HPO, http://human-phenotype-ontology.github.io/, entering at Jan 2018) - a database for collecting information on genetic phenotypes in human disease [18]. (4) Online Mendelian Inheritance in Man (OMIM, http://omim.org/, entering at Jan 2018) - a database for collecting comprehensive compendium of human genes [19]. (5) The Comparative Toxicogenomics Database (CTD, http://ctdbase.org/, entering at Jan 2018) - a database for collecting phenotype human disease [20]. The latter three databases are all authoritative databases which collect pneumonia-related phenotype. We retrieved these databases with a keyword “pneumonia” and obtained 51,136 genes in total.

#### Protein-protein interaction data

PPI data on 296 common targets between YD and pneumonia were extracted from STRING (http://string-db.org/, v. 10) with parameters filtered by “*Homo sapiens*” (confidence score > 0.4), which is a database covering no less than 2000 organisms and constructing interaction network between proteins by novel algorithms [21]. Subsequently, the data description of the correlation between every two targets, defined as the score, was acquired.

### Topological analysis of YD-pneumonia target network

Data from STRING were input into Cytoscape version 3.6.0 for topological analysis. Cytoscape is designed to visualize the network of biological pathways and intermolecular interaction networks. Moreover, topological parameters can be measured by Network Analyzer, a plug-in of Cytoscape, for complicated network analysis [22]. Finally, we obtained 103 hub targets with 3 key topological parameters as a degree, closeness and betweenness centralities larger than the median, and matched them with the candidate components. Thus, 68 bioactive ingredients were correlated with 103 hub targets from YD and pneumonia. The PPI analysis was performed as described above.

### Network construction

First, we built an interaction network of the related herbs and bioactive components in YD. Then, the interaction between potential common YD-related targets and pneumonia-related targets were obtained based on PPI data and topological analysis. Finally, a holistic network of herbs, components, and targets of YD-pneumonia was constructed.

### Network analysis

#### Cluster

In protein interaction networks, topological modules or clusters represent dense regions of highly correlated molecular components in pure network property. The clusters were extracted by calculating the corresponding networks with Molecular Complex Detection. Finally, we obtained 4 clusters of highly interconnected targets.

#### Gene ontology and KEGG pathway enrichment

Clusters were input into The Database for Annotation, Visualization and Integrated Discovery (DAVID, https://david-d.ncifcrf.gov/, version 6.7) [23] for Gene ontology (GO) and Kyoto Encyclopedia of Genes and Genomes (KEGG, https://www.kegg.jp/, entering at Jan 2018) for pathway enrichment analysis. Several pneumonia-related biological processes and pathways were returned. The biological processes and pathways with Bonferroni< 0.05 were considered to be significant. To further uncover the therapeutic mechanism of YD, we identified 8 pathways associated with pneumonia mechanism.

### Experiment validation

#### Animals and models

Eighteen Clean grade Sprague-Dawley *(*SD) rats, male, 4-weeks-old, weighing 110 ± 10 g, were purchased from Sibeifu (Beijing) Biotechnology Co., Ltd., license No.: SCXK (Jing) 2016–0002 (animal certificate No.: 1103241800000595). They were raised in the animal room of the Beijing University of traditional Chinese medicine with free access to food and water, natural light. The rats were randomly divided into three groups: normal control group (NC), pneumonia group (P), pneumonia treatment group (PT), 6 rats in each group. Rats were adaptively bred for 3 days before starting the experiment. From the first day of formal experiment, all groups were given ordinary feed. P group was sprayed with 5 ml LPS solution (0.5 mg/ml) one time a day for 30 min. NC group and PT group were given equal dose of pure water atomization. The rats in PT group were administrated YD (2 ml/100 g) by means of intragastric administration contrasting with normal saline (2 ml/100 g) given to rats in NC group and P group. After 3 days’ model building, all the rats were fast but free for water. On the fourth morning, the rats were anesthetized by intraperitoneal injection of 10% chloral hydrate (0.2 ml/100 g). The left lung was removed and placed in a 4% formaldehyde fixative, and stored at 4 °C, while the right lung placed in a frozen pipe and frozen by liquid nitrogen, then stored at − 80 °C.

#### Chemical drug and agents

Lipopolysaccharides (LPS from Eschericia coli 055: B5, 100 mg, 046m4045v, sigma company, USA). ELISA Kit for Interleukin 6 (IL6) (SEA079Ra, 96 t, Cloud-Clone Corp., USA).

#### Tissue detection

The transverse sections of the left lung tissues were prepared according to the routine procedure for tissue wax block-slice-hematoxylin-eosin (HE) staining. The right lung tissue homogenate was prepared and detected by ELISA method according to instruction.

## Results

### Herb-component network of YD

**Fig. 2:**
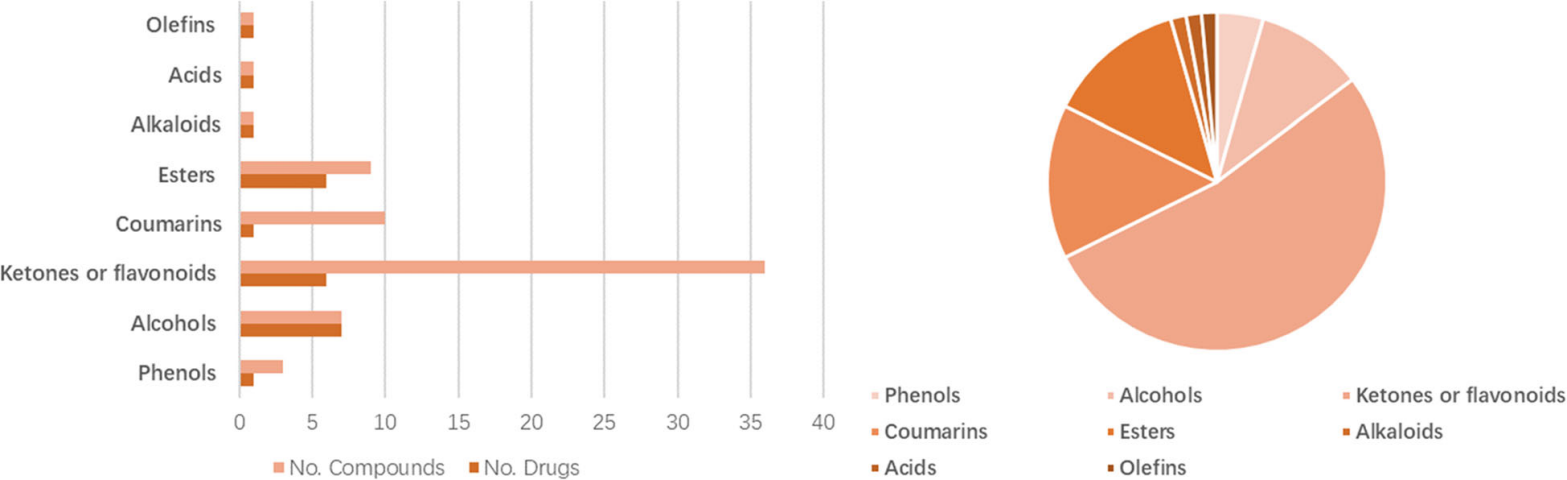
Number and percentage of different active compounds of YD

**Fig. 3:**
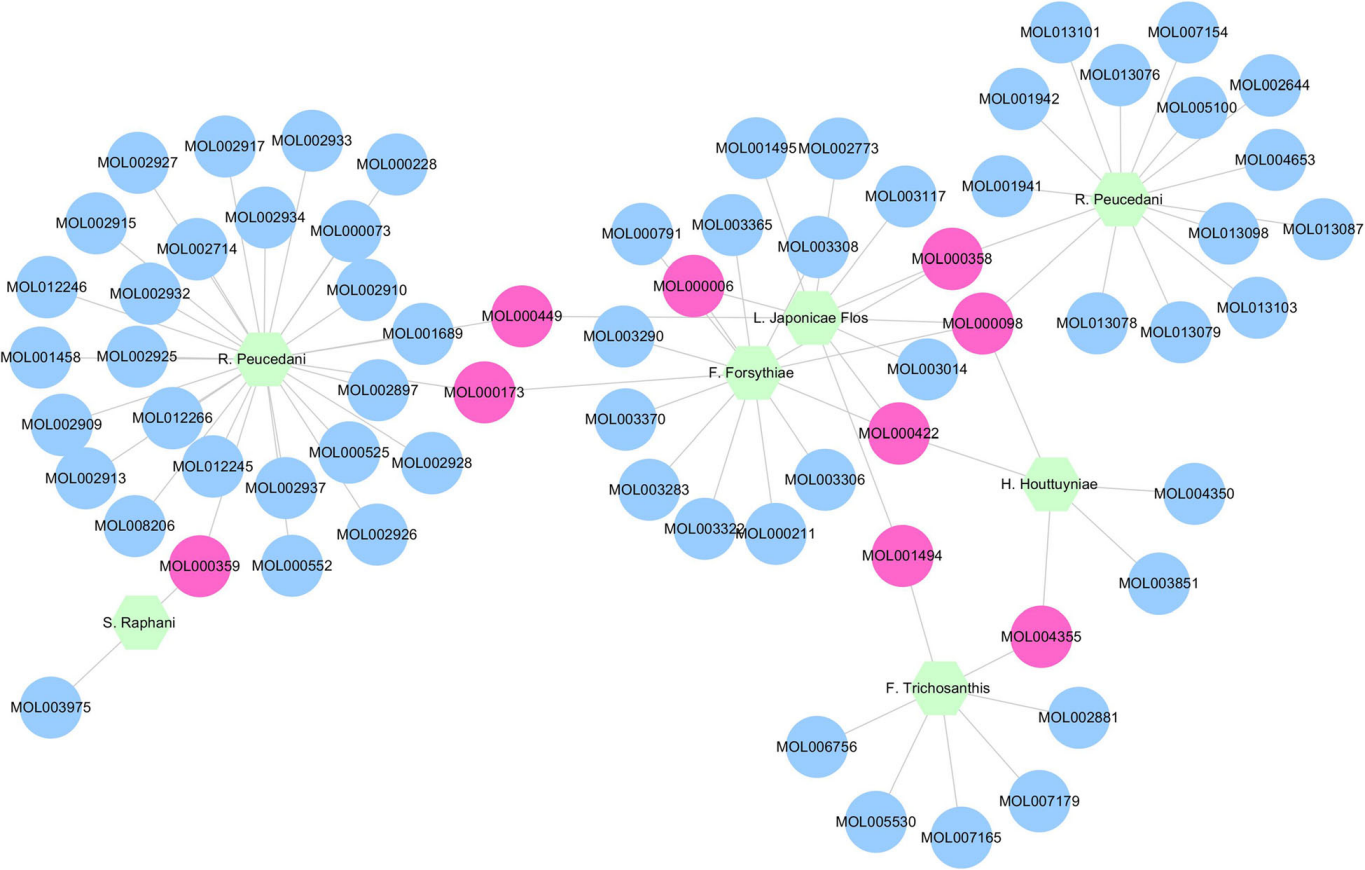
Herb-component network of YD. Each gray edge represents the functional relationship between the two nodes it connects. Blue ellipse represents active components related to YD and pneumonia; pink ellipse represents active components related to more than one herb in YD; green hexagon represents the herbs of YD

Generally, herbs are administered through oral route and eventually enter the blood circulation where they are maintained at a certain concentration gradient that has a pharmacological effect on the patient [24]. After administration, the concentration of the bioactive ingredients at the target site is controlled by the pharmacokinetic process, which ultimately affects the therapeutic response [25]. Bearing this in mind, we opted for three classical ADME properties which were OB, Caco-2 and DL to filter the bioactive components of YD. Sixty-eight components related to pneumonia and having good ADME properties were obtained (as shown in Supplementary Information Table S1) from database search. The top-listed compounds were ketones/flavonoids (52.94%), followed by coumarins (14.71%), esters (13.24%), alcohols (10.29%), phenols (4.41%), alkaloids (1.47%), acids (1.47%), and olefins (1.47%) (as displayed in the Fig. 2). Nine components were involved in more than one herb, including luteolin, quercetin, wogonin, beta-sitosterol, sitosterol, kaempferol, mandenol, stigmasterol and spinasterol, 8 of which belong to ketones or flavonoids. The connection was established between components and herbs in YD (as displayed in Fig. 3).

### YD-pneumonia target network

**Fig. 4:**
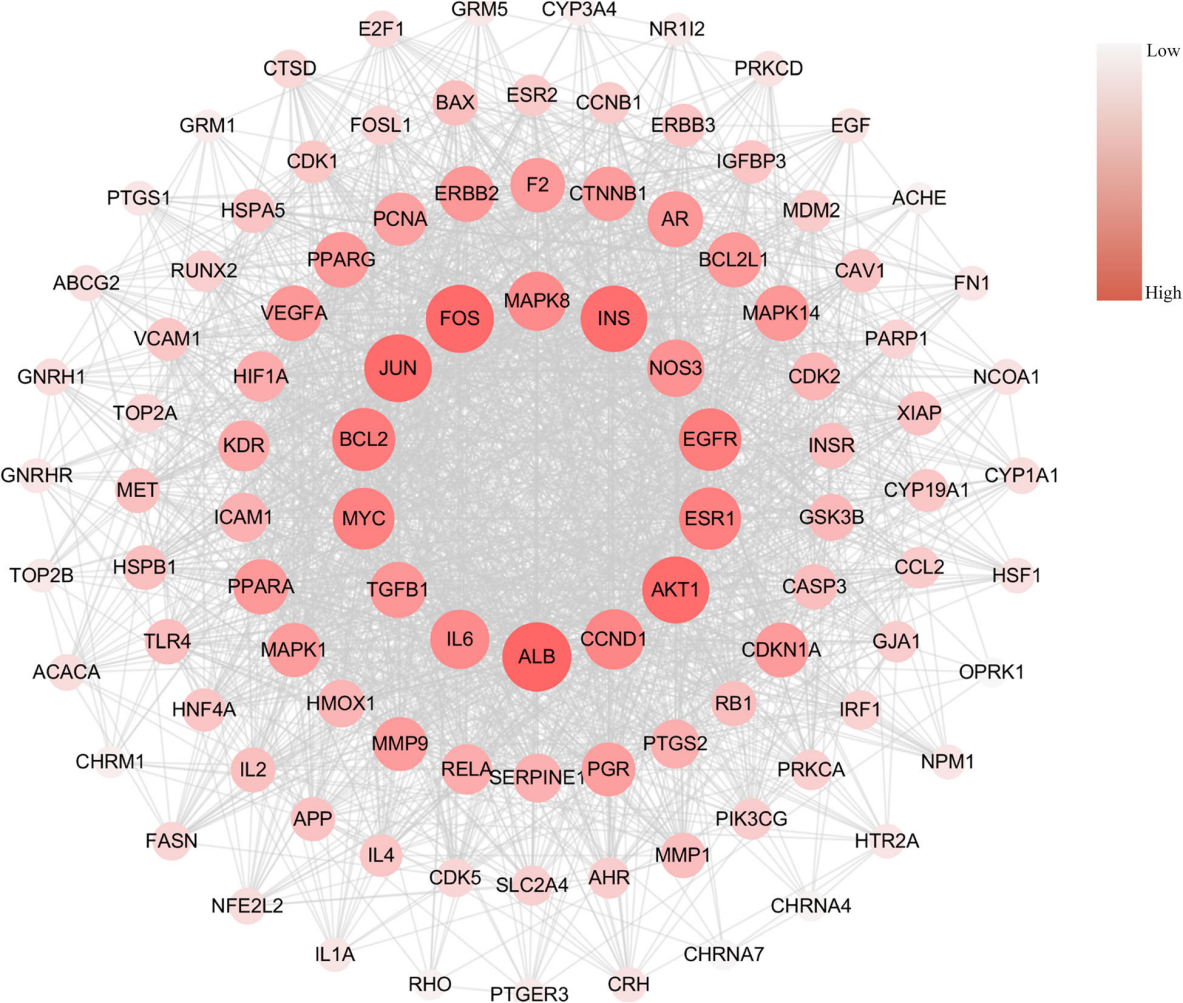
YD-pneumonia target network. Each gray edge represents the functional relationship between the two nodes it connects. Each red ellipse represents targets related to both YD and pneumonia. The size and color depth of nodes are positively correlated with their degrees, respectively

The topological analysis was performed to uncover the properties of the proteins and to explore key therapeutic targets of YD. There are different features to represent the position of the nodes in an interaction network, such as average shortest path length, betweenness centrality, closeness centrality, clustering coefficient, degree and topological coefficient. Among them, degree, closeness, and betweenness centrality are used to evaluate the centrality of nodes in the network [26]. It is an important characteristic of large-scale networks because degree is the number of connections to a node and closeness is used to measure the degree of centralization. The node with higher closeness centrality goes through a shorter way to the center. Betweenness centrality shows how frequently a node flows on all shortest paths. As the betweenness centrality is higher, the more important a node is [27–29]. We defined nodes with the degree, closeness, and betweenness centrality all above the median as hub targets, which occupies the central position of the network. We searched “pneumonia” as a keyword in Therapeutic Target Database (TTD), Human Phenotype Ontology (HPO), Online Mendelian Inheritance in Man (OMIM), The Comparative Toxicogenomics Database (CTD), and finally obtained 51,136 related targets, and 296 of them overlapping to YD related targets. After topological eigenvalue calculation, we selected 103 hub targets and 1897 edges (as shown in Supplementary Information Table S2). Finally, we constructed a target-target network based on the overlapping data between YD-related targets and pneumonia-related targets, indicating potential pharmacological functions in YD for treating pneumonia (as displayed in Fig. 4).

### Herb-component-target of YD-pneumonia network

**Fig. 5:**
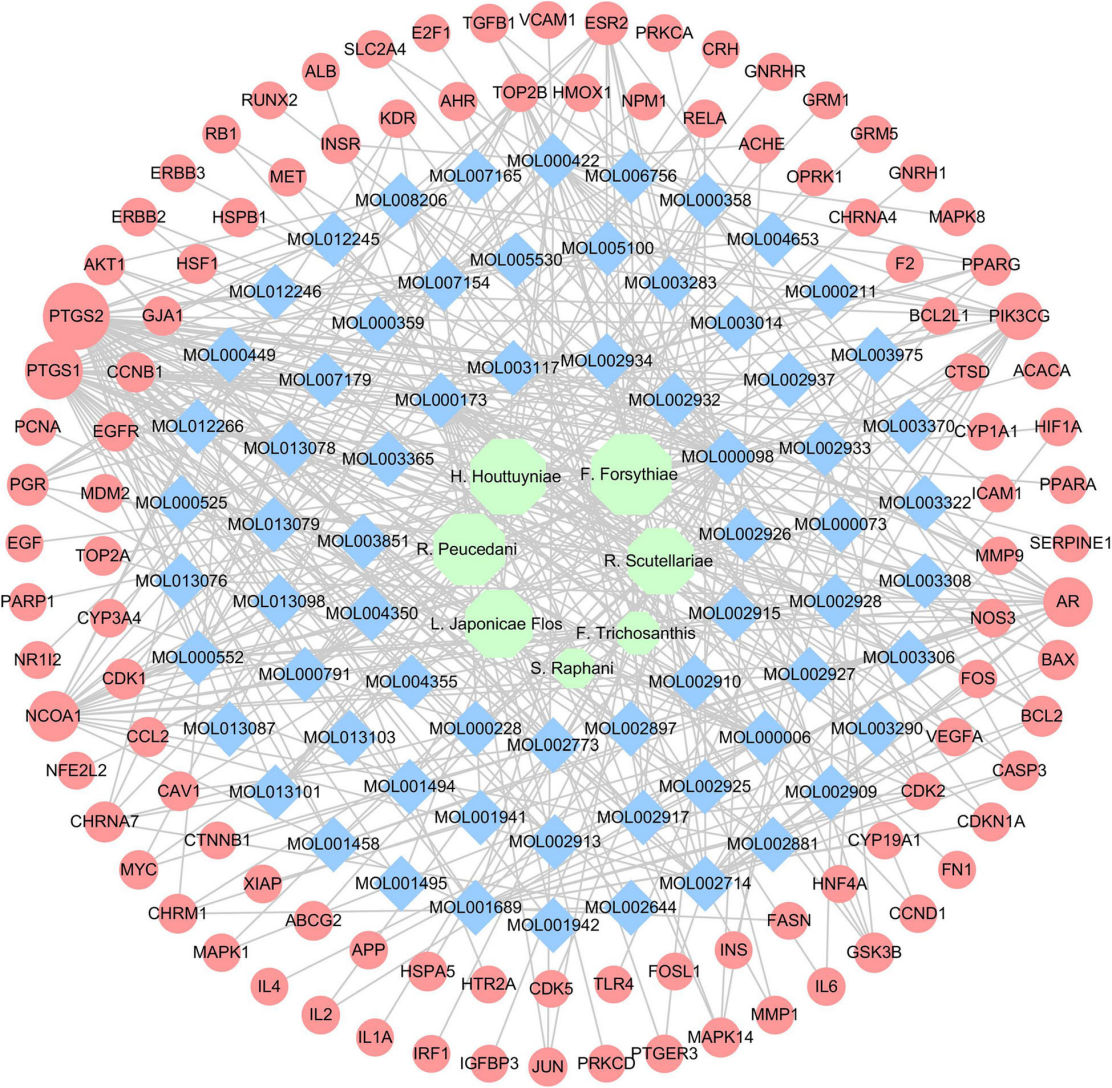
Herb-component-target network Green hexagon represents the herbs of YD; blue diamond represents components of YD; red ellipse represents YD-pneumonia targets, respectively. The gray lines represent the relationship among herbs, components, and targets. The size of nodes is positively correlated with their degrees

We constructed an “herb-component-target of YD-pneumonia” network. It showed that an herb could interact with multiple components, and a component could also target several targets related to pneumonia (as shown in Supplementary Information Table S3). In the sequence of importance, the herbs in YD were as follows: LQ (*F. suspensa*, degree = 67), YXC (*H. cordata Thunb*, degree = 59), QH (*P. praeruptorum Dunn*, degree = 53), JYH (*L. japonica Thunb*, degree = 47), HQ (*S. baicalensis Georgi*, degree = 45), GL (*F. rosthornii Harms*, degree = 8), and LFZ (*R. sativus L*, degree = 4). Arranged by the importance, the targets were as follows: prostaglandin G/H synthase 1 (PTGS1, degree = 32), prostaglandin G/H synthase 2 (PTGS2, degree = 46), nuclear receptor coactivator 1 (NCOA1, degree = 21), androgen receptor (AR, degree = 19), phosphatidylinositol 4,5-bisphosphate 3-kinase catalytic subunit gamma (PIK3CG, degree = 16), estrogen receptor 2 (ESR2, degree = 10) and so forth. It demonstrated that the ingredients of YD might play a “multi-targets and multi-components” therapeutic effect in the intervention of pneumonia-related targets (as displayed in Fig. 5).

### YD-pneumonia network analysis

**Fig. 6:**
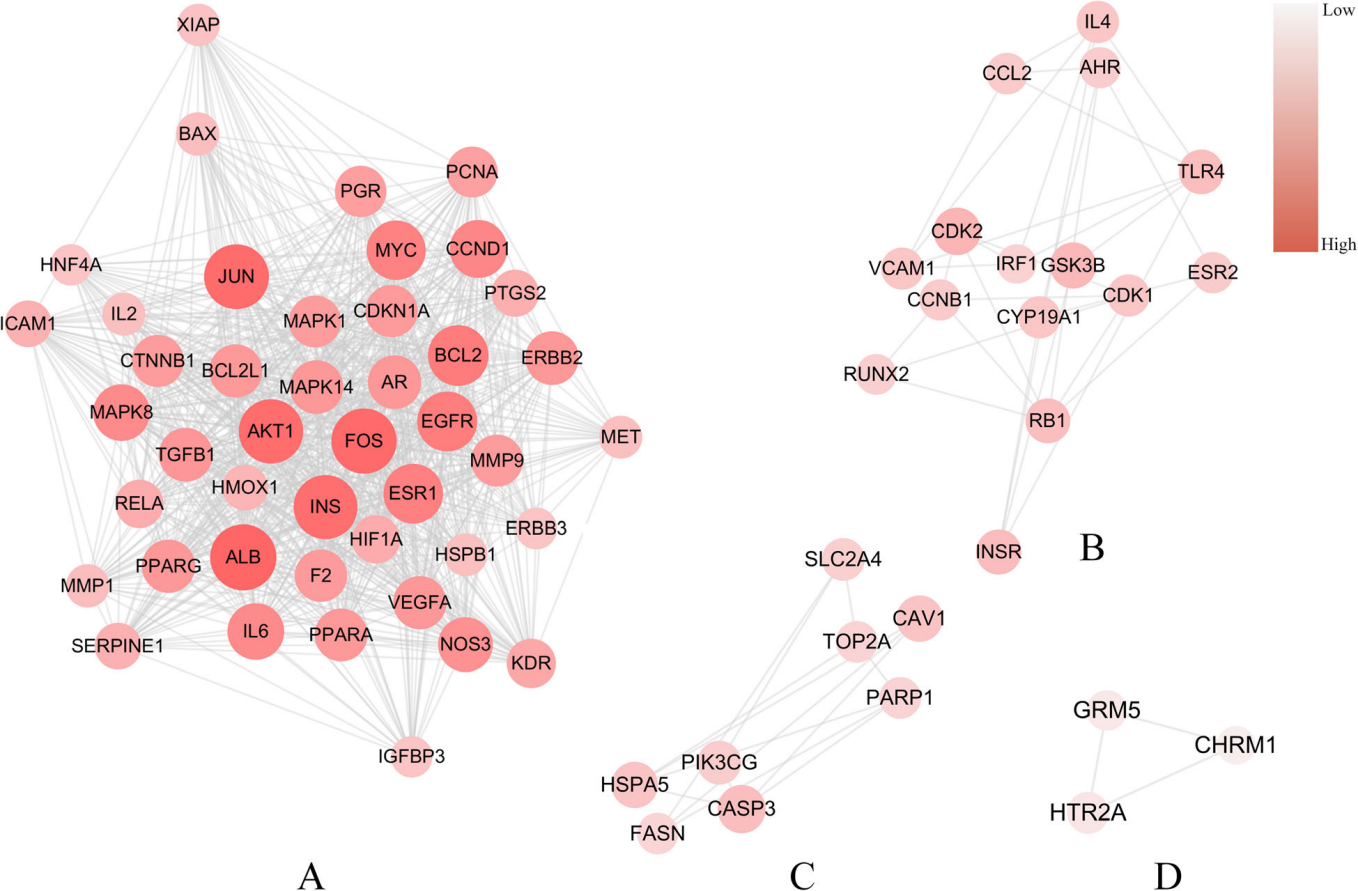
Four clusters of hub targets. Each gray edge represents the functional relationship between the two nodes it connects. Each ellipse represents a target protein related to YD and pneumonia. The size and color depth of nodes are positively correlated with their degrees, respectively

**Fig. 7:**
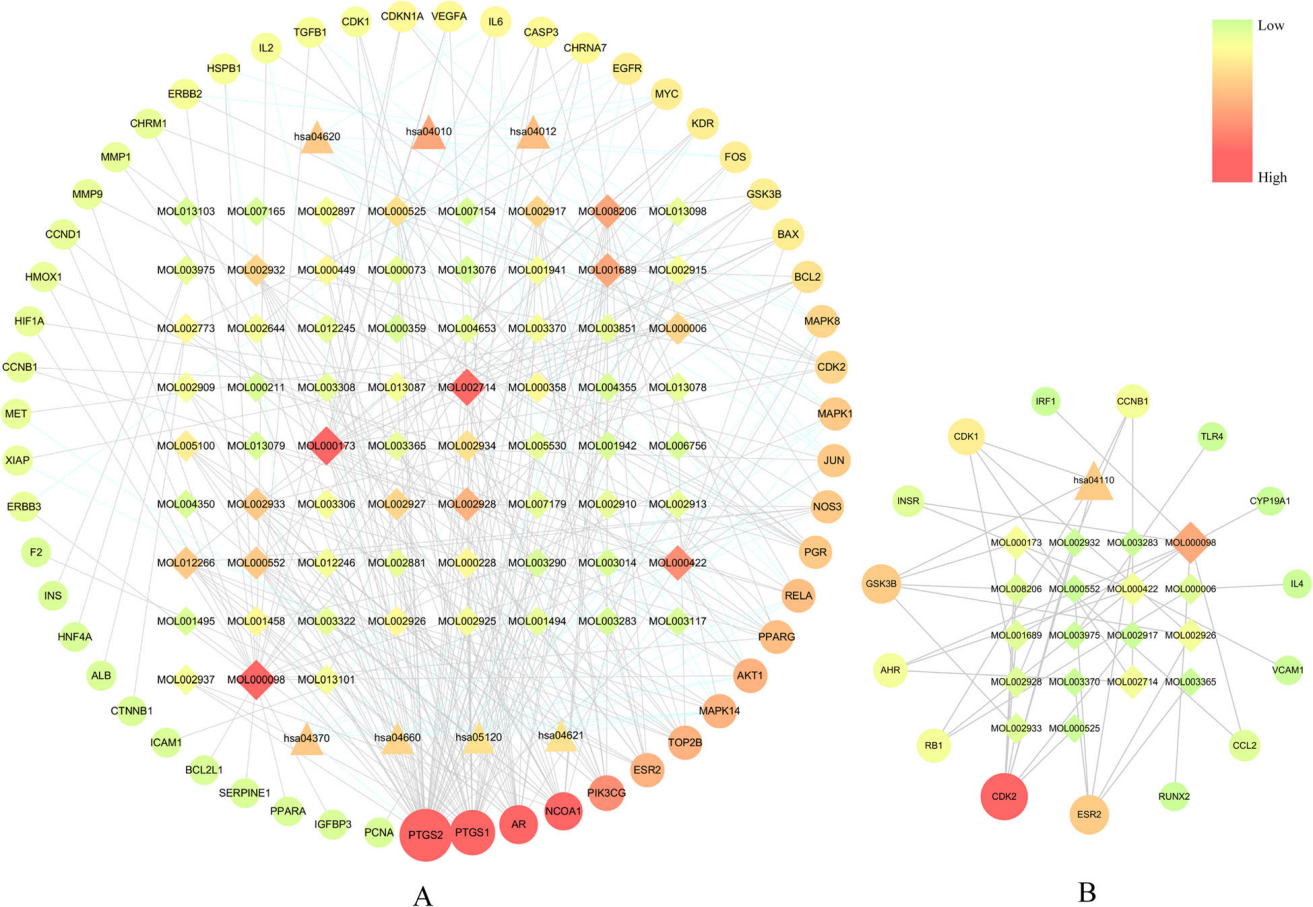
Target-component-pathway network of clusters A and B. Triangles stand for pathways, diamonds for components, and ellipses for targets, respectively. The gray lines represent the relationship among herbs, components, and targets. The size and color depth of nodes are positively correlated with their degrees

**Fig. 8:**
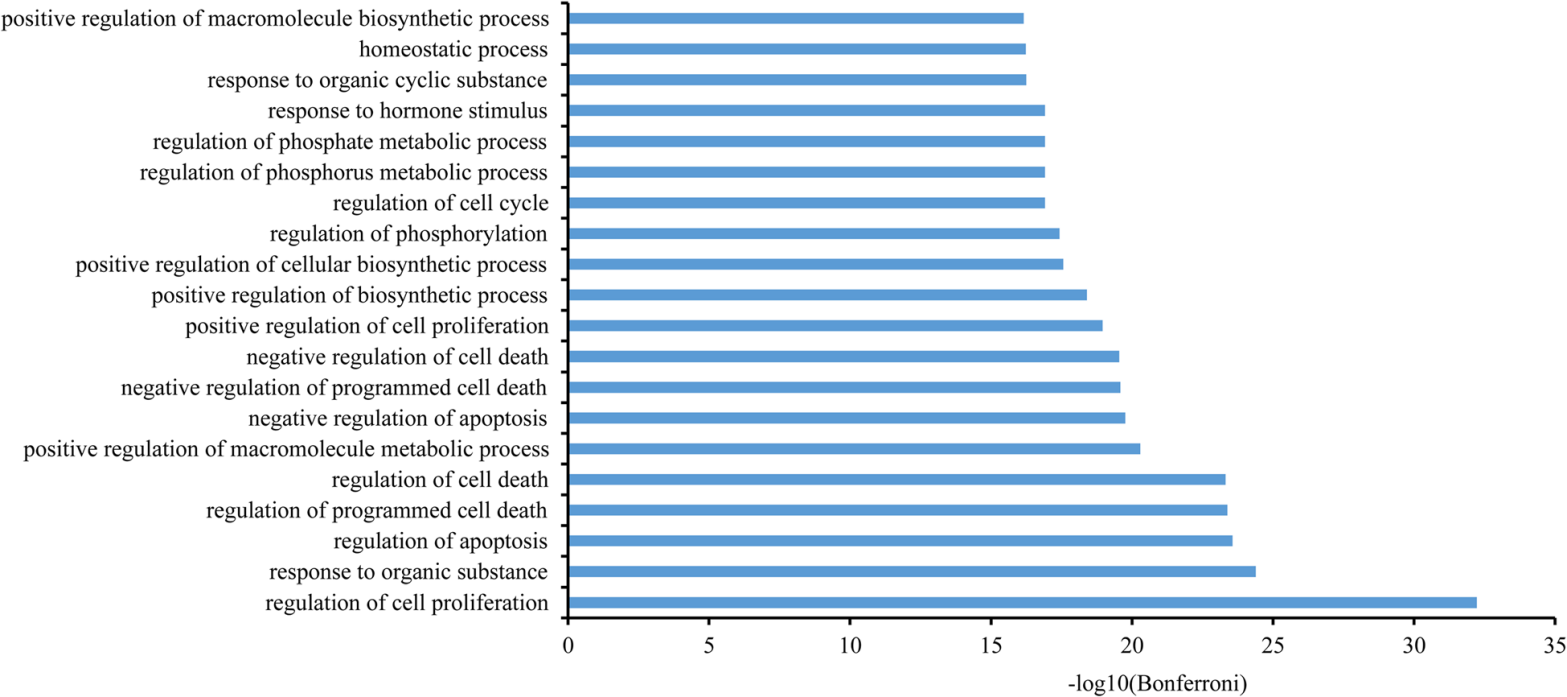
GO biological processes of YD for pneumonia. The top 20 biological processes are presented (Bonferroni< 0.05). It mainly involves the processes relevant to cell proliferation, apoptosis and death and metabolism of energy and substances

We analyzed the network with a cluster search algorithm, which considers highly connected dense regions as a network (Molecular Complex Detection), and four clusters were returned (as displayed in Fig. 6). Then, clusters were input into DAVID and KEGG for GO and pathway enrichment analysis, and only clusters A and B were involved in biological processes and pathways associated with pneumonia. According to the pathogenesis of pneumonia, these biological processes and pathways could be divided into three modules: (1) immunity and inflammation; (2) angiogenesis and epithelial cell proliferation; (3) cell growth, proliferation and apoptosis (as displayed in the Figs. 7 and 8).

#### Immunity and inflammation

Several biological processes associated with immunity and inflammation were returned and showed as follows: (GO:0002520) immune system development, (GO:0002684) positive regulation of immune system process, (GO:0002697) regulation of immune effector process, (GO:0006954) inflammatory response, (GO:0006954) inflammatory response, (GO:0001817) regulation of cytokine production, (GO:0042110) T cell activation, (GO:0002521) leukocyte differentiation, (GO:0001776) leukocyte homeostasis, (GO:0032496) response to lipopolysaccharide and so forth (Bonferroni< 0.05). Additionally, three high-degree pathways were captured to be closely related to immunity and inflammation such as (hsa04620) Toll-like receptor signaling pathway (degree = 8), (hsa04621) NOD-like receptor signaling pathway (degree = 6), and (hsa04660) T cell receptor signaling pathway (degree = 7).

#### Angiogenesis and epithelial cell proliferation

In our work, some biological processes related with angiogenesis and epithelial cell proliferation were returned such as (GO:0001568) blood vessel development, (GO:0001944) vasculature development, (GO:0048514) blood vessel morphogenesis, (GO:0050678) regulation of epithelial cell proliferation, (GO:0050679) positive regulation of epithelial cell proliferation, (GO:0001525) angiogenesis and (GO:0045765) regulation of angiogenesis (Bonferroni< 0.05). Additionally, three high-degree pathways, (hsa04012) ErbB signaling pathway (degree = 9), (hsa04370) VEGF signaling pathway (degree = 8), and (hsa05120) epithelial cell signaling in *Helicobacter pylori* infection (degree = 6) were detected.

#### Cell growth, proliferation, and apoptosis

A variety of biological processes associated with cell growth, proliferation and apoptosis were returned, such as (GO:0042127) regulation of cell proliferation, (GO:0042981) regulation of apoptosis, (GO:0010941) regulation of cell death, (GO:0043067) regulation of programmed cell death, (GO:0043066) negative regulation of apoptosis, (GO:0043069) negative regulation of programmed cell death, (GO:0060548) negative regulation of cell death, (GO:0006916) anti-apoptosis and so forth (Bonferroni< 0.05). Additionally, two high-degree pathways, (hsa04010) MAPK signaling pathway (degree = 11) and (hsa04110) Cell cycle (degree = 5) were detected.

### Experiment validation

#### HE staining of lung tissue pathology

**Fig. 9:**
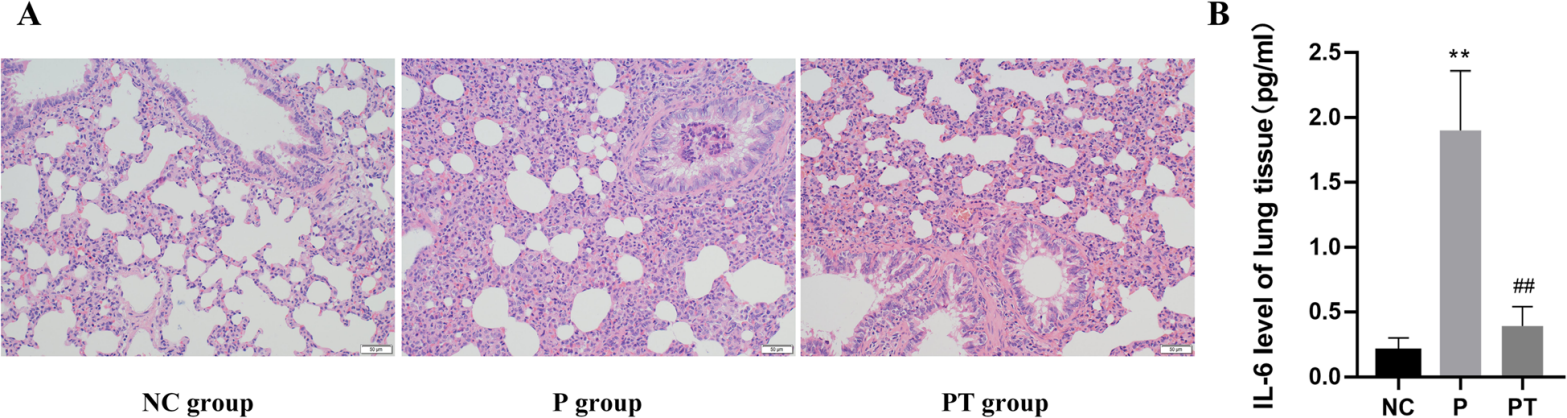
Pathology and cytokines validation of lung tissue. **a**. Pathology of lung tissues by method of HE staining (200X). **b**. The cytokine level of IL-6 in lung tissue. Values are represented as means ± SEM, *n* = 6 per group. *P < 0.05, **P < 0.01versus NC group, #*P* < 0.05, ##*P* < 0.01 versus P group

According to the results of HE staining of lung tissue pathology, the structure of alveoli in NC group was intact and thin, without inflammatory infiltration, and the structure of bronchioles was intact and clear. Compared with NC group, the lung tissue structure in P group was damaged, and alveolar septum size and alveolar wall were thick and fragile. There was a large number of neutrophil infiltration and red cell extravasation in the lung interstation in P group. The structure of lung tissue and alveoli in PT group was clearer than that of P group, and the growth of pulmonary interstitial was reduced, with a small amount of inflammatory infiltration (as displayed in Fig. 9a).

#### Cytokines of lung tissue

The level of IL-6 of lung tissue was detected, which was significant higher in P group than NC group (*P* < 0.01). After YD treatment, the level of IL-6 in the lung tissue decreased significantly (*P* < 0.01) (as displayed in Fig. 9b).

## Discussion

One of the common respiratory diseases is pneumonia. Due to drug resistance and undesired effects, limited options are currently available for its treatment. YD is a traditional Chinese medicine and extensively used in Chinese clinics for the treatment of pneumonia, mainly in children. However, its detailed mechanism of action is not discovered yet and thus attempted to explore in this network pharmacological study.

### Anti-pathogenic and anti-inflammatory effects of YD ingredients

This study revealed that YD had a large proportion of flavonoids such as luteolin, quercetin, wogonin, and kaempferol. Flavonoids possess a variety of pharmacological effects including antibacterial, anti-virus and anti-inflammatory [30]. Researchers have found that desaminotyrosine (DAT) is a microbial metabolite of flavonoids, which can regulate type I interferon (IFN) signaling pathway and reduce lung pathological response for influenza treatment [31]. Immunomodulatory effects of wogonin on viral infection, lipopolysaccharides (LPS) stimulation and the imbalance of Th1/Th2/Th17 were observed [32]. Wogonin also directly attenuated the enzymatic activity of cyclooxygenase-2 (COX-2) at a low concentration [33]. The ability to inhibit nitric oxide synthase (NOS), COX-2 and C- reactive protein (CRP) protein was positively correlated with all concentrations of Kaempferol, but the inhibition effect of quercetin at high concentration was decreased [34]. This fact indicated that YD might exert anti-bacterial, anti-viral and anti-inflammatory activities in a certain dose-dependent manner.

### Two features of YD

This study showed a multi-target feature of YD since 61 out of its 103 targets were related with several other targets (not less than 30), making a network and suggesting that the combined effects of multiple targets was responsible for the pharmacodynamics of YD. YD might modulate multiple systems to achieve the treatment of pneumonia. As shown in the network, hub targets of YD for treating pneumonia are not only concentrated on the modulation of the immune inflammatory response but also focused on the regulation of other accompanying factors related to cell proliferation, stress-activated cell survival, vascular system, and endocrine system [35–37].

### The underlying mechanisms of YD in treating pneumonia

**Fig. 10:**
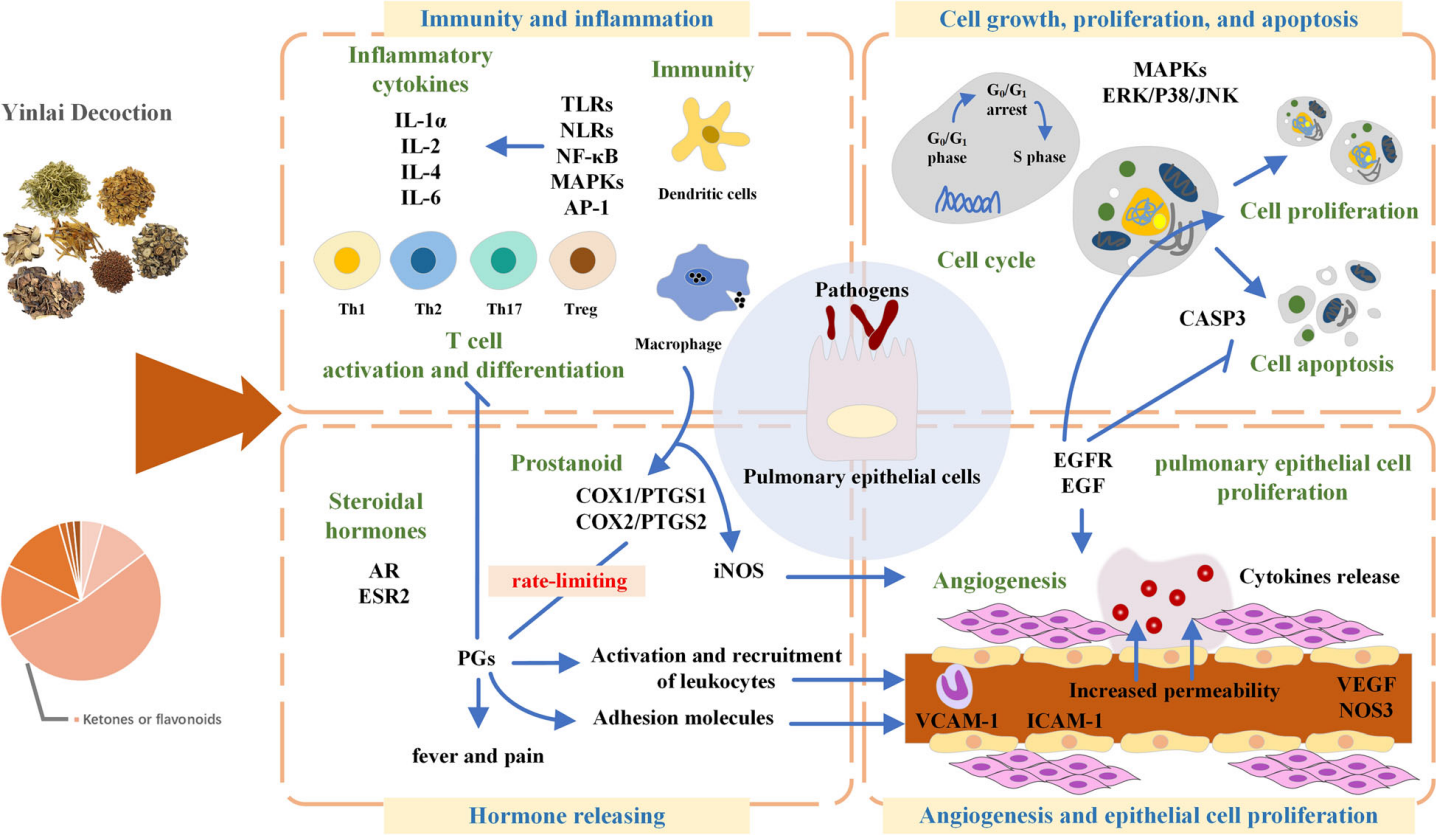
Different mechanisms involved in the YD treatment of pneumonia. The triangular arrow represents activation, and the slanted arrow means inhibition. The activation and inhibition mechanisms showed in this figure are based on the knowledge from the literature and are not obtained from the results of any of the used platforms

TCM-based treatment system deals with the treatment of an individual (or a system) as a whole from an integrative and holistic way, in contrast to the “single-component and single-target” mode of the Western drugs. The drug resistance and side effects of Western drugs for pneumonia create an increasing trend of TCM therapy. Network pharmacology provides the researchers with a good paradigm. In this study, YD-pneumonia related targets and pathways were assembled to decipher the underlying mechanisms of YD in treating pneumonia from a systematic perspective. Since several targets can affect different pathways synchronously, resulting in a cross-regulation of multi-target and multi-pathway, current functional clusters were extracted and analyzed to assess the potential mechanisms associated with pneumonia (as displayed in Fig. 10).

### Relieving clinical symptoms of pneumonia

The active components of YD have potential interaction with a variety of targets like PTGS1 and PTGS2, which are implicated in the mechanisms of multiple inflammatory diseases, including lung injury [38, 39]. The pulmonary inflammatory reaction is modulated by regulating prostaglandins (PGs) release that is controlled by respiratory epithelial cells. Cyclooxygenase (COX) is a key rate-limiting enzyme in PG biosynthesis. COX1 (PTGS1) is involved in the early stage of PGs physiological synthesis, whereas COX-2 (PTGS2), as the inducible isoform of COX, persists for a few hours after stimulation by an inflammatory response. Thus, YD might be involved in the alleviation of inflammatory reaction, fever, and pain by inhibiting PG synthesis.

### Regulation of hormone release

It is noteworthy that several targets regulated by the herbs in YD like androgen receptor (AR) and ESR2, are implicated in the endocrine system. There is an increasing evidence that sex hormones possess pathophysiological actions in the lung. The gender-related difference in the morbidity and mortality of respiratory diseases has been gradually noted in clinical practice [40]. Previous studies have reported that exposure to cigarette smoke results in a higher likeliness of suffering from lung disorders with an up-regulated expression of AR contributing to a series of downstream pathological changes. It further explained the association between sex hormones and pulmonary diseases [41] and may act as another mechanism for the treatment of pneumonia by YD.

### Regulation of host immune inflammatory response

It is a potential mechanism involved in the YD effect on the immune system. The recognition of pathogen-associated molecular patterns (PAMPs) by phagocytic cells and dendritic cells (DCs) is the first step to activate the host’s immune system, ultimately contributing to the activation of intracellular signaling pathways and adaptive immune system. For the past few years, it has been convinced that the immune system primely perceives the environmental information after infection through Toll-like receptors (TLRs) expressed on the membrane of antigen-presenting cells (APCs) recognizing invasive pathogens. There are 10 different kinds of TLRs up to now [42, 43]. Because of TLRs’ limited distribution, NOD-like receptors (NLRs) can play an anti-infection role as intracellular receptors that respond to antigens. Therefore, NLRS can provide an identification system that may play a key role in the reaction of microbes or its cell wall components, which have already entered the cell or have escaped the surveillance of TLRs [44]. The APCs activated by TLR initiate innate and adaptive immune systems and promote the differentiation of T-cells to different directions of Th1, Th2, Th17 and Treg, which are closely related to the occurrence of various respiratory diseases such as pneumonia, influenza and asthma [45]. YD may treat pneumonia through regulating more than one pathway, which confirms that disorders are treated by using TCM in an integrative and holistic way.

### Intervention on angiogenesis and epithelial cell proliferation

Several targets in EGFR, epidermal growth factor receptor (ErbB) and vascular endothelial growth factor (VEGF) signaling pathways involved in angiogenesis and epithelial cell proliferation are predicted in this study. The airway epithelial barrier defenses against the inhaled antigens and pathogens. EGFR plays a significant role in angiogenesis and the barrier function of the airway epithelium against pathogens, and simultaneously, lung epithelium TLRs expression initiate immune responses through triggering EGFR downstream signal cascade [46]. VEGF plays an important role in angiogenesis and maintenance of vascular permeability, which is one of the characteristic findings contributing to the respiratory tract remodeling. *Staphylococcus aureus* (*S. aureus*) and *Streptococcus pneumoniae* (*S. pneumoniae*) are two common factors for serious and complicated pneumonia. Researchers found that *S. aureus* and *S. pneumoniae* stimulated the release of VEGF from normal mesothelial cells and human neutrophils in a dose-dependent and time-dependent manner, respectively [47, 48]. In addition, chronic inflammation and hyperimmune response are common characteristics of several lung diseases associated with *Helicobacter pylori* (Hp) infection, including chronic bronchitis, asthma, and lung cancer. Researchers found that Hp-infected patients might suffer from an increased risk of pneumonia for high gastric pH levels caused by Hp infection and gastric acid inhibitor [49]. Above all, the results indicate that YD is effective in the treatment of pneumonia through regulating angiogenesis and epithelial cell proliferation.

### Regulation of cell cycle arrest and apoptosis

Several potential targets, such as JNK1 (MAPK8), ERK2 (MAPK1) and p38-α (MAPK14) participate in mitogen-activated protein kinase (MAPK) pathways which act as a regulator of cell cycle arrest and apoptosis [50] and play an important role in signal transduction during the inflammatory reaction [51]. As we all know, the replication of the virus occurs by regulating the cell cycle progression of the host, which is a feature of their pathogenic mechanism. As a common pathogeny of pediatric pneumonia and bronchitis, an efficient replication of the respiratory syncytial virus (RSV) can be ensured by mediating cell cycle arrest via increasing transforming growth factor-β (TGF-β) and MAPK signaling pathway [52]. Thus, the results display another potential mechanism that YD may target MAPK signaling pathways and cell cycle to inhibit respiratory viral replication and signal transduction during the inflammatory response.

### The systematic role of YD in the treatment of pneumonia

In summary, the results of network construction and analysis demonstrate that YD, as a multi-target agent, comprises multiple components and alleviates symptoms of pneumonia through regulating host immune inflammatory response (like TLRs, nuclear factor-kappa B (NF-κB) and IL-6), angiogenesis and vascular permeability [such as VEGF, NOS3, vascular cell adhesion molecule-1 (VCAM − 1) and intercellular adhesion molecule 1 (ICAM-1)], barrier function of the airway epithelial cells (like EGFR and EGF), hormone-releasing (such as AR, ESR2, PTGS1, and PTGS2), and the pivotal proteins in the process of cell cycle and signal transduction (such as JNK, ERK and p38). These biological processes and pathways are not isolated, but connected, interacting with each other to target pneumonia.

### Limitation of network pharmacology

There are still a few unknown compounds and their targets, thus the pharmacological effects of an herbal formula cannot be revealed completely. Another limitation is the false negative due to the targets rooted from a different database, which may have biased impact because of different experimental conditions. We are also aware of the public databases that have limited information. At present, some public databases tend to focus on some hot researches, so complete pharmacological effects of some other disease cannot be revealed.

### Experimental verification

Network pharmacology showed that IL-6 was a remarkable predicted target which was related with infection. In the inflammatory reaction, the rise of IL-6 was earlier than other cytokines, and lasted for a long time, so it can be used to assist the early diagnosis of acute infection. After bacterial infection, the level of IL-6 increased rapidly and reached the peak at 2 h, which was consistent with the severity of infection. Therefore, IL-6 has more advantages as an inflammatory marker. It showed that YD could significantly alleviate the inflammatory changes in the lung and reduce the expression of IL-6 in the lung tissue.

## Conclusion

Upon the above findings, we assume that YD may not directly target pathogens but exerts its therapeutic effect against pneumonia by adjusting the pulmonary environment and overall state of the host. YD turns out to be a potential “multi-component and multi-target” agent, providing a novel approach for clinical practice. However, our work only dissects some potential therapeutic mechanisms of YD and a small amount of verification work has been carried out. Further experimental verification is still necessary for the future.

## Supplementary information

**Additional file 1:Table S1.** Active ingredients and ADME parameters of YD. **Table S2**. Target information of related active ingredients. **Table S3**. The relationship between active ingredients and potential targets.

## Data Availability

The datasets generated and/or analyzed during the current study are available from the corresponding author on reasonable request.

## Abbreviations

DL: Druglikeness
GL: Trichosanthes rosthornii Harms
IL: Interleukin
HE: Hematoxylin-eosin
HQ: Scutellaria baicalensis Georgi
JYH: *Lonicera japonica* Thunb
LFZ: *Raphanus sativus* L
LQ: *Forsythia suspensa* (Thunb.) Vahl
NC: Normal contral
OB: Oral Bioavailability
P: Pneumonia
PPI: Protein-protein interaction
PT: Pneumonia tratment
QH: Peucedanum praeruptorum Dunn
TCM: Traditional Chinese medicine
YD: Yinlai Decoction
YXC: *Houttuynia cordata* Thunb
