# Supplementary material for: Network pharmacology to dissect the mechanisms of Yinlai Decoction for pneumonia
Source: BMC Complement Med Ther. 2020 Jun 3;20:168. doi: 10.1186/s12906-020-02954-z (PMC7267769; doi:10.1186/s12906-020-02954-z)
Supplement: Supplementary file 1 — Additional file 1:Table S1. Active ingredients and ADME parameters of YD. Table S2. Target information of related active ingredients. Table S3. The relationship between active ingredients and potential targets. [file 12906_2020_2954_MOESM1_ESM.docx]

**Network pharmacology to dissect the mechanisms of Yinlai Decoction for pneumonia**

Jingnan Xu2^,+^, Chen Bai^1,+^, Ling Huang^1,+^, Tiegang Liu^1, +^, Yuxiang Wan^2^, Zian Zheng^3^, Xueyan Ma^1^, Fei Gao^1^, He Yu^1^, Xiaohong Gu^1,^*

Table S1 Active ingredients and ADME parameters of YD

| MOL ID | Molecule Name | OB | Caco-2 | DL | Herbs |
| --- | --- | --- | --- | --- | --- |
| MOL000006 | luteolin | 36.16 | 0.19 | 0.25 | Lonicerae Japonicae Flos Forsythiae Fructus |
| MOL000073 | ent-Epicatechin | 48.96 | 0.02 | 0.24 | Scutellariae Radix |
| MOL000098 | quercetin | 46.43 | 0.05 | 0.28 | Lonicerae Japonicae Flos Forsythiae Fructus  Scutellariae Radix  Peucedani Radix  Houttuyniae Herba |
| MOL000173 | wogonin | 30.68 | 0.79 | 0.23 | Forsythiae Fructus  Scutellariae Radix |
| MOL000211 | Mairin | 55.38 | 0.73 | 0.78 | Forsythiae Fructus |
| MOL000228 | (2R)-7-hydroxy-5-methoxy-2-phenylchroman-4-one | 55.23 | 0.87 | 0.2 | Scutellariae Radix |
| MOL000358 | beta-sitosterol | 36.91 | 1.32 | 0.75 | Lonicerae Japonicae Flos Forsythiae Fructus  Peucedani Radix |
| MOL000359 | sitosterol | 36.91 | 1.32 | 0.75 | Scutellariae Radix  Raphani Semen |
| MOL000422 | kaempferol | 41.88 | 0.26 | 0.24 | Lonicerae Japonicae Flos Forsythiae Fructus  Houttuyniae Herba |
| MOL000449 | Stigmasterol | 43.83 | 1.44 | 0.76 | Lonicerae Japonicae Flos |
| MOL000525 | Norwogonin | 39.4 | 0.6 | 0.21 | Scutellariae Radix |
| MOL000552 | 5,2'-Dihydroxy-6,7,8-trimethoxyflavone | 31.71 | 0.93 | 0.35 | Scutellariae Radix |
| MOL000791 | bicuculline | 69.67 | 0.72 | 0.88 | Forsythiae Fructus |
| MOL001458 | coptisine | 30.67 | 1.21 | 0.86 | Scutellariae Radix |
| MOL001494 | Mandenol | 42 | 1.46 | 0.19 | Lonicerae Japonicae Flos Trichosanthes Kirilowii Maxim |
| MOL001495 | Ethyl linolenate | 46.1 | 1.54 | 0.2 | Lonicerae Japonicae Flos |
| MOL001689 | acacetin | 34.97 | 0.67 | 0.24 | Scutellariae Radix |
| MOL001941 | Ammidin | 34.55 | 1.13 | 0.22 | Peucedani Radix |
| MOL001942 | isoimperatorin | 45.46 | 0.97 | 0.23 | Peucedani Radix |
| MOL002644 | Phellopterin | 40.19 | 0.98 | 0.28 | Peucedani Radix |
| MOL002714 | baicalein | 33.52 | 0.63 | 0.21 | Scutellariae Radix |
| MOL002773 | beta-carotene | 37.18 | 2.25 | 0.58 | Lonicerae Japonicae Flos |
| MOL002881 | Diosmetin | 31.14 | 0.46 | 0.27 | Trichosanthes Kirilowii Maxim |
| MOL002897 | epiberberine | 43.09 | 1.17 | 0.78 | Scutellariae Radix |
| MOL002909 | 5,7,2,5-tetrahydroxy-8,6-dimethoxyflavone | 33.82 | 0.35 | 0.45 | Scutellariae Radix |
| MOL002910 | Carthamidin | 41.15 | 0.16 | 0.24 | Scutellariae Radix |
| MOL002913 | Dihydrobaicalin_qt | 40.04 | 0.56 | 0.21 | Scutellariae Radix |
| MOL002915 | Salvigenin | 49.07 | 0.86 | 0.33 | Scutellariae Radix |
| MOL002917 | 5,2',6'-Trihydroxy-7,8-dimethoxyflavone | 45.05 | 0.48 | 0.33 | Scutellariae Radix |
| MOL002925 | 5,7,2',6'-Tetrahydroxyflavone | 37.01 | 0.18 | 0.24 | Scutellariae Radix |
| MOL002926 | dihydrooroxylin A | 38.72 | 0.71 | 0.23 | Scutellariae Radix |
| MOL002927 | Skullcapflavone II | 69.51 | 0.68 | 0.44 | Scutellariae Radix |
| MOL002928 | oroxylin a | 41.37 | 0.76 | 0.23 | Scutellariae Radix |
| MOL002932 | Panicolin | 76.26 | 0.84 | 0.29 | Scutellariae Radix |
| MOL002933 | 5,7,4'-Trihydroxy-8-methoxyflavone | 36.56 | 0.46 | 0.27 | Scutellariae Radix |
| MOL002934 | NEOBAICALEIN | 104.34 | 0.74 | 0.44 | Scutellariae Radix |
| MOL002937 | DIHYDROOROXYLIN | 66.06 | 0.67 | 0.23 | Scutellariae Radix |
| MOL003014 | secologanic dibutylacetal_qt | 53.65 | 0.34 | 0.29 | Lonicerae Japonicae Flos |
| MOL003117 | Ioniceracetalides B_qt | 61.19 | -0.09 | 0.19 | Lonicerae Japonicae Flos |
| MOL003283 | (2R,3R,4S)-4-(4-hydroxy-3-methoxy-phenyl)-7-methoxy-2,3-dimethylol-tetralin-6-ol | 66.51 | -0.2 | 0.39 | Forsythiae Fructus |
| MOL003290 | (3R,4R)-3,4-bis[(3,4-dimethoxyphenyl) methyl] oxolan-2-one | 52.3 | 0.78 | 0.48 | Forsythiae Fructus |
| MOL003306 | ACon1_001697 | 85.12 | 0.76 | 0.57 | Forsythiae Fructus |
| MOL003308 | (+)-pinoresinol monomethyl ether-4-D-beta-glucoside_qt | 61.2 | 0.7 | 0.57 | Forsythiae Fructus |
| MOL003322 | FORSYTHINOL | 81.25 | 0.59 | 0.57 | Forsythiae Fructus |
| MOL003365 | Lactucasterol | 40.99 | 0.88 | 0.85 | Forsythiae Fructus |
| MOL003370 | Onjixanthone I | 79.16 | 0.84 | 0.3 | Forsythiae Fructus |
| MOL003851 | Isoramanone | 39.97 | 0.05 | 0.51 | Houttuyniae Herba |
| MOL003975 | icosa-11,14,17-trienoic acid methyl ester | 44.81 | 1.52 | 0.23 | Raphani Semen |
| MOL004350 | Ruvoside_qt | 36.12 | -0.31 | 0.76 | Houttuyniae Herba |
| MOL004355 | Spinasterol | 42.98 | 1.44 | 0.76 | Houttuyniae Herba Trichosanthes Kirilowii Maxim |
| MOL004653 | (+)-Anomalin | 46.06 | 0.46 | 0.66 | Scutellariae Radix |
| MOL005100 | 5,7-dihydroxy-2-(3-hydroxy-4-methoxyphenyl) chroman-4-one | 47.74 | 0.28 | 0.27 | Scutellariae Radix |
| MOL005530 | Hydroxygenkwanin | 36.47 | 0.52 | 0.27 | Trichosanthes Kirilowii Maxim |
| MOL006756 | Schottenol | 37.42 | 1.33 | 0.75 | Trichosanthes Kirilowii Maxim |
| MOL007154 | tanshinone iia | 49.89 | 1.05 | 0.4 | Peucedani Radix |
| MOL007165 | 10α-cucurbita-5,24-diene-3β-ol | 44.02 | 1.46 | 0.74 | Trichosanthes Kirilowii Maxim |
| MOL007179 | Linolenic acid ethyl ester | 46.1 | 1.48 | 0.2 | Trichosanthes Kirilowii Maxim |
| MOL008206 | Moslosooflavone | 44.09 | 1.01 | 0.25 | Scutellariae Radix |
| MOL012245 | 5,7,4'-trihydroxy-6-methoxyflavanone | 36.63 | 0.43 | 0.27 | Scutellariae Radix |
| MOL012246 | 5,7,4'-trihydroxy-8-methoxyflavanone | 74.24 | 0.37 | 0.26 | Scutellariae Radix |
| MOL012266 | rivularin | 37.94 | 0.65 | 0.37 | Scutellariae Radix |
| MOL013076 | (8S,9R)-9-hydroxy-8-(2-hydroxypropan-2-yl)-8,9-dihydrofuro[2,3-h] chromen-2-one | 37.3 | 0.1 | 0.2 | Peucedani Radix |
| MOL013078 | praeruptorin E | 51.22 | 0.37 | 0.66 | Peucedani Radix |
| MOL013079 | dl-praeruptorin a | 46.46 | 0.52 | 0.53 | Peucedani Radix |
| MOL013087 | Peucedanocoumarin II | 63.48 | 0.25 | 0.53 | Peucedani Radix |
| MOL013098 | [(9R)-8,8-dimethyl-2-oxo-9,10-dihydropyrano[6,5-h] chromen-9-yl] (Z)-2-methylbut-2-enoate | 87.48 | 0.61 | 0.37 | Peucedani Radix |
| MOL013101 | rutarin_qt | 70.1 | 0.2 | 0.2 | Peucedani Radix |
| MOL013103 | 532-16-1 | 46.57 | 1.43 | 0.44 | Peucedani Radix |

Table S2 Target information of related active ingredients.

| Entry | Target name | gene name | Degree |
| --- | --- | --- | --- |
| [Q9UNQ0](http://www.uniprot.org/uniprot/Q9UNQ0) | ATP-binding cassette sub-family G member 2 | ABCG2 | 19 |
| [Q13085](http://www.uniprot.org/uniprot/Q13085) | Acetyl-CoA carboxylase 1 | ACACA | 20 |
| [P22303](http://www.uniprot.org/uniprot/P22303) | Acetylcholinesterase | ACHE | 10 |
| [P35869](http://www.uniprot.org/uniprot/P35869) | Aryl hydrocarbon receptor | AHR | 29 |
| [P31749](http://www.uniprot.org/uniprot/P31749) | RAC-alpha serine/threonine-protein kinase | AKT1 | 81 |
| [P02768](http://www.uniprot.org/uniprot/P02768) | Serum albumin | ALB | 85 |
| [P05067](http://www.uniprot.org/uniprot/P05067) | Amyloid beta A4 protein | APP | 35 |
| [P10275](http://www.uniprot.org/uniprot/P10275) | Androgen receptor | AR | 58 |
| [Q07812](http://www.uniprot.org/uniprot/Q07812) | Apoptosis regulator BAX | BAX | 37 |
| [P10415](http://www.uniprot.org/uniprot/P10415) | Apoptosis regulator Bcl-2 | BCL2 | 73 |
| [Q07817](http://www.uniprot.org/uniprot/Q07817) | Bcl-2-like protein 1 | BCL2L1 | 57 |
| [P42574](http://www.uniprot.org/uniprot/P42574) | Caspase-3 | CASP3 | 37 |
| [Q03135](http://www.uniprot.org/uniprot/Q03135) | Caveolin-1 | CAV1 | 34 |
| [P13500](http://www.uniprot.org/uniprot/P13500) | C-C motif chemokine 2 | CCL2 | 30 |
| [P14635](http://www.uniprot.org/uniprot/P14635) | G2/mitotic-specific cyclin-B1 | CCNB1 | 30 |
| [P24385](http://www.uniprot.org/uniprot/P24385) | G1/S-specific cyclin-D1 | CCND1 | 67 |
| [P06493](http://www.uniprot.org/uniprot/P06493) | Cell division control protein 2 homolog | CDK1 | 32 |
| [P24941](http://www.uniprot.org/uniprot/P24941) | Cell division protein kinase 2 | CDK2 | 42 |
| [Q00535](http://www.uniprot.org/uniprot/Q00535) | Cell division protein kinase 5 | CDK5 | 24 |
| [P38936](http://www.uniprot.org/uniprot/P38936) | Cyclin-dependent kinase inhibitor 1 | CDKN1A | 56 |
| [P11229](http://www.uniprot.org/uniprot/P11229) | Muscarinic acetylcholine receptor M1 | CHRM1 | 11 |
| [P43681](http://www.uniprot.org/uniprot/P43681) | Neuronal acetylcholine receptor subunit alpha-4 | CHRNA4 | 8 |
| [P36544](http://www.uniprot.org/uniprot/P36544) | Neuronal acetylcholine receptor subunit alpha-7 | CHRNA7 | 7 |
| [P06850](http://www.uniprot.org/uniprot/P06850) | Corticoliberin | CRH | 18 |
| [P35222](http://www.uniprot.org/uniprot/P35222) | Catenin beta-1 | CTNNB1 | 55 |
| [P07339](http://www.uniprot.org/uniprot/P07339) | Cathepsin D | CTSD | 23 |
| [P11511](http://www.uniprot.org/uniprot/P11511) | Cytochrome P450 19A1 | CYP19A1 | 32 |
| [P04798](http://www.uniprot.org/uniprot/P04798) | Cytochrome P450 1A1 | CYP1A1 | 20 |
| [P08684](http://www.uniprot.org/uniprot/P08684) | Cytochrome P450 3A4 | CYP3A4 | 12 |
| [Q01094](http://www.uniprot.org/uniprot/Q01094) | Transcription factor E2F1 | E2F1 | 22 |
| [P01133](http://www.uniprot.org/uniprot/P01133) | Pro-epidermal growth factor | EGF | 17 |
| [P00533](http://www.uniprot.org/uniprot/P00533) | Epidermal growth factor receptor | EGFR | 72 |
| [P04626](http://www.uniprot.org/uniprot/P04626) | Receptor tyrosine-protein kinase erbB-2 | ERBB2 | 58 |
| [P21860](http://www.uniprot.org/uniprot/P21860) | Receptor tyrosine-protein kinase erbB-3 | ERBB3 | 33 |
| [P03372](http://www.uniprot.org/uniprot/P03372) | Estrogen receptor | ESR1 | 71 |
| [Q92731](http://www.uniprot.org/uniprot/Q92731) | Estrogen receptor beta | ESR2 | 30 |
| [P00734](http://www.uniprot.org/uniprot/P00734) | Prothrombin | F2 | 56 |
| [P49327](http://www.uniprot.org/uniprot/P49327) | Fatty acid synthase | FASN | 24 |
| [P02751](http://www.uniprot.org/uniprot/P02751) | Fibronectin | FN1 | 16 |
| [P01100](http://www.uniprot.org/uniprot/P01100) | Proto-oncogene c-Fos | FOS | 83 |
| [P21525](http://www.uniprot.org/uniprot/P21525) | Fos-related antigen 1 | FOSL1 | 26 |
| [P17302](http://www.uniprot.org/uniprot/P17302) | Gap junction alpha-1 protein | GJA1 | 29 |
| [P01148](http://www.uniprot.org/uniprot/P01148) | Progonadoliberin-1 | GNRH1 | 20 |
| [P30968](http://www.uniprot.org/uniprot/P30968) | Gonadotropin-releasing hormone receptor | GNRHR | 16 |
| [Q13255](http://www.uniprot.org/uniprot/Q13255) | Metabotropic glutamate receptor 1 | GRM1 | 12 |
| [P41594](http://www.uniprot.org/uniprot/P41594) | Metabotropic glutamate receptor 5 | GRM5 | 14 |
| [P49841](http://www.uniprot.org/uniprot/P49841) | Glycogen synthase kinase-3 beta | GSK3B | 40 |
| [Q16665](http://www.uniprot.org/uniprot/Q16665) | Hypoxia-inducible factor 1-alpha | HIF1A | 47 |
| [P09601](http://www.uniprot.org/uniprot/P09601) | Heme oxygenase 1 | HMOX1 | 42 |
| [P41235](http://www.uniprot.org/uniprot/P41235) | Hepatocyte nuclear factor 4-alpha | HNF4A | 33 |
| [Q00613](http://www.uniprot.org/uniprot/Q00613) | Heat shock factor protein 1 | HSF1 | 18 |
| [P11021](http://www.uniprot.org/uniprot/P11021) | 78 kDa glucose-regulated protein | HSPA5 | 34 |
| [P04792](http://www.uniprot.org/uniprot/P04792) | Heat shock protein beta-1 | HSPB1 | 36 |
| [P28223](http://www.uniprot.org/uniprot/P28223) | 5-hydroxytryptamine 2A receptor | HTR2A | 16 |
| [P05362](http://www.uniprot.org/uniprot/P05362) | Intercellular adhesion molecule 1 | ICAM1 | 44 |
| [P17936](http://www.uniprot.org/uniprot/P17936) | Insulin-like growth factor-binding protein 3 | IGFBP3 | 33 |
| [P01583](http://www.uniprot.org/uniprot/P01583) | Interleukin-1 alpha | IL1A | 15 |
| [P60568](http://www.uniprot.org/uniprot/P60568) | Interleukin-2 | IL2 | 36 |
| [P05112](http://www.uniprot.org/uniprot/P05112) | Interleukin-4 | IL4 | 32 |
| [P05231](http://www.uniprot.org/uniprot/P05231) | Interleukin-6 | IL6 | 65 |
| [P01308](http://www.uniprot.org/uniprot/P01308) | Insulin | INS | 80 |
| [P06213](http://www.uniprot.org/uniprot/P06213) | Insulin receptor | INSR | 38 |
| [P10914](http://www.uniprot.org/uniprot/P10914) | Interferon regulatory factor 1 | IRF1 | 26 |
| [P05412](http://www.uniprot.org/uniprot/P05412) | Transcription factor AP-1 | JUN | 82 |
| [P35968](http://www.uniprot.org/uniprot/P35968) | Vascular endothelial growth factor receptor 2 | KDR | 49 |
| [P28482](http://www.uniprot.org/uniprot/P28482) | Mitogen-activated protein kinase 1 | MAPK1 | 55 |
| [Q16539](http://www.uniprot.org/uniprot/Q16539) | Mitogen-activated protein kinase 14 | MAPK14 | 58 |
| [P45983](http://www.uniprot.org/uniprot/P45983) | Mitogen-activated protein kinase 8 | MAPK8 | 65 |
| [Q00987](http://www.uniprot.org/uniprot/Q00987) | E3 ubiquitin-protein ligase Mdm2 | MDM2 | 30 |
| [P08581](http://www.uniprot.org/uniprot/P08581) | Hepatocyte growth factor receptor | MET | 36 |
| [P03956](http://www.uniprot.org/uniprot/P03956) | Interstitial collagenase | MMP1 | 37 |
| [P14780](http://www.uniprot.org/uniprot/P14780) | Matrix metalloproteinase-9 | MMP9 | 55 |
| [P01106](http://www.uniprot.org/uniprot/P01106) | Myc proto-oncogene protein | MYC | 70 |
| [Q15788](http://www.uniprot.org/uniprot/Q15788) | Nuclear receptor coactivator 1 | NCOA1 | 18 |
| [Q16236](http://www.uniprot.org/uniprot/Q16236) | Nuclear factor erythroid 2-related factor 2 | NFE2L2 | 20 |
| [P29474](http://www.uniprot.org/uniprot/P29474) | Nitric oxide synthase, endothelial | NOS3 | 61 |
| [P06748](http://www.uniprot.org/uniprot/P06748) | Nucleophosmin | NPM1 | 16 |
| [O75469](http://www.uniprot.org/uniprot/O75469) | Nuclear receptor subfamily 1 group I member 2 | NR1I2 | 13 |
| [P41145](http://www.uniprot.org/uniprot/P41145) | Kappa-type opioid receptor | OPRK1 | 6 |
| [P09874](http://www.uniprot.org/uniprot/P09874) | Poly [ADP-ribose] polymerase 1 | PARP1 | 25 |
| [P12004](http://www.uniprot.org/uniprot/P12004) | Proliferating cell nuclear antigen | PCNA | 53 |
| [P06401](http://www.uniprot.org/uniprot/P06401) | Progesterone receptor | PGR | 54 |
| [P48736](http://www.uniprot.org/uniprot/P48736) | Phosphatidylinositol-4,5-bisphosphate 3-kinase catalytic subunit, gamma isoform | PIK3CG | 29 |
| [Q07869](http://www.uniprot.org/uniprot/Q07869) | Peroxisome proliferator-activated receptor alpha | PPARA | 57 |
| [P37231](http://www.uniprot.org/uniprot/P37231) | Peroxisome proliferator activated receptor gamma | PPARG | 58 |
| [P17252](http://www.uniprot.org/uniprot/P17252) | Protein kinase C alpha type | PRKCA | 28 |
| [Q05655](http://www.uniprot.org/uniprot/Q05655) | Protein kinase C delta type | PRKCD | 17 |
| [P43115](http://www.uniprot.org/uniprot/P43115) | Prostaglandin E2 receptor EP3 subtype | PTGER3 | 13 |
| [P23219](http://www.uniprot.org/uniprot/P23219) | Prostaglandin G/H synthase 1 | PTGS1 | 16 |
| [P35354](http://www.uniprot.org/uniprot/P35354) | Prostaglandin G/H synthase 2 | PTGS2 | 45 |
| [P06400](http://www.uniprot.org/uniprot/P06400) | Retinoblastoma-associated protein | RB1 | 37 |
| [Q04206](http://www.uniprot.org/uniprot/Q04206) | Transcription factor p65 | RELA | 47 |
| [P08100](http://www.uniprot.org/uniprot/P08100) | Rhodopsin | RHO | 9 |
| [Q13950](http://www.uniprot.org/uniprot/Q13950) | Runt-related transcription factor 2 | RUNX2 | 28 |
| [P05121](http://www.uniprot.org/uniprot/P05121) | Plasminogen activator inhibitor 1 | SERPINE1 | 44 |
| [P14672](http://www.uniprot.org/uniprot/P14672) | Solute carrier family 2, facilitated glucose transporter member 4 | SLC2A4 | 27 |
| [P01137](http://www.uniprot.org/uniprot/P01137) | Transforming growth factor beta-1 | TGFB1 | 59 |
| [O00206](http://www.uniprot.org/uniprot/O00206) | Toll-like receptor 4 | TLR4 | 37 |
| [P11388](http://www.uniprot.org/uniprot/P11388) | DNA topoisomerase 2-alpha | TOP2A | 25 |
| [E9PCY5](http://www.uniprot.org/uniprot/E9PCY5) | DNA topoisomerase II | TOP2B | 14 |
| [P19320](http://www.uniprot.org/uniprot/P19320) | Vascular cell adhesion protein 1 | VCAM1 | 32 |
| [P15692](http://www.uniprot.org/uniprot/P15692) | Vascular endothelial growth factor A | VEGFA | 58 |
| [P98170](http://www.uniprot.org/uniprot/P98170) | Baculoviral IAP repeat-containing protein 4 | XIAP | 35 |

Table S3 The relationship between active ingredients and potential targets.

| Mol ID | Molecule name | Target name | gene |
| --- | --- | --- | --- |
| MOL000006 | luteolin | Amyloid beta A4 protein | APP |
| MOL000006 | luteolin | Baculoviral IAP repeat-containing protein 4 | XIAP |
| MOL000006 | luteolin | DNA topoisomerase 2-alpha | TOP2A |
| MOL000006 | luteolin | E3 ubiquitin-protein ligase Mdm2 | MDM2 |
| MOL000006 | luteolin | Epidermal growth factor receptor | EGFR |
| MOL000006 | luteolin | G2/mitotic-specific cyclin-B1 | CCNB1 |
| MOL000006 | luteolin | Hepatocyte growth factor receptor | MET |
| MOL000006 | luteolin | Interleukin-2 | IL2 |
| MOL000006 | luteolin | Interleukin-4 | IL4 |
| MOL000006 | luteolin | Mitogen-activated protein kinase 1 | MAPK1 |
| MOL000006 | luteolin | Proliferating cell nuclear antigen | PCNA |
| MOL000006 | luteolin | Receptor tyrosine-protein kinase erbB-2 | ERBB2 |
| MOL000006 | luteolin | Retinoblastoma-associated protein | RB1 |
| MOL000073 | ent-Epicatechin | Prostaglandin G/H synthase 1 | PTGS1 |
| MOL000073 | ent-Epicatechin | Prostaglandin G/H synthase 2 | PTGS2 |
| MOL000098 | quercetin | Interleukin-1 alpha | IL1A |
| MOL000098 | quercetin | Myc proto-oncogene protein | MYC |
| MOL000098 | quercetin | Aryl hydrocarbon receptor | AHR |
| MOL000098 | quercetin | Cell division control protein 2 homolog | CDK1 |
| MOL000098 | quercetin | Heat shock protein beta-1 | HSPB1 |
| MOL000098 | quercetin | Heme oxygenase 1 | HMOX1 |
| MOL000098 | quercetin | Insulin receptor | INSR |
| MOL000098 | quercetin | Peroxisome proliferator activated receptor gamma | PPARG |
| MOL000098 | quercetin | RAC-alpha serine/threonine-protein kinase | AKT1 |
| MOL000098 | quercetin | Solute carrier family 2, facilitated glucose transporter member 4 | SLC2A4 |
| MOL000098 | quercetin | 78 kDa glucose-regulated protein | HSPA5 |
| MOL000098 | quercetin | Acetyl-CoA carboxylase 1 | ACACA |
| MOL000098 | quercetin | ATP-binding cassette sub-family G member 2 | ABCG2 |
| MOL000098 | quercetin | Bcl-2-like protein 1 | BCL2L1 |
| MOL000098 | quercetin | Cathepsin D | CTSD |
| MOL000098 | quercetin | Caveolin-1 | CAV1 |
| MOL000098 | quercetin | C-C motif chemokine 2 | CCL2 |
| MOL000098 | quercetin | Cyclin-dependent kinase inhibitor 1 | CDKN1A |
| MOL000098 | quercetin | DNA topoisomerase 2-alpha | TOP2A |
| MOL000098 | quercetin | Epidermal growth factor receptor | EGFR |
| MOL000098 | quercetin | G1/S-specific cyclin-D1 | CCND1 |
| MOL000098 | quercetin | G2/mitotic-specific cyclin-B1 | CCNB1 |
| MOL000098 | quercetin | Gap junction alpha-1 protein | GJA1 |
| MOL000098 | quercetin | Heat shock factor protein 1 | HSF1 |
| MOL000098 | quercetin | Hypoxia-inducible factor 1-alpha | HIF1A |
| MOL000098 | quercetin | Insulin-like growth factor-binding protein 3 | IGFBP3 |
| MOL000098 | quercetin | Interferon regulatory factor 1 | IRF1 |
| MOL000098 | quercetin | Interleukin-2 | IL2 |
| MOL000098 | quercetin | Matrix metalloproteinase-9 | MMP9 |
| MOL000098 | quercetin | Mitogen-activated protein kinase 1 | MAPK1 |
| MOL000098 | quercetin | Nitric oxide synthase, endothelial | NOS3 |
| MOL000098 | quercetin | Nuclear factor erythroid 2-related factor 2 | NFE2L2 |
| MOL000098 | quercetin | Peroxisome proliferator-activated receptor alpha | PPARA |
| MOL000098 | quercetin | Plasminogen activator inhibitor 1 | SERPINE1 |
| MOL000098 | quercetin | Poly [ADP-ribose] polymerase 1 | PARP1 |
| MOL000098 | quercetin | Pro-epidermal growth factor | EGF |
| MOL000098 | quercetin | Prostaglandin E2 receptor EP3 subtype | PTGER3 |
| MOL000098 | quercetin | Proto-oncogene c-Fos | FOS |
| MOL000098 | quercetin | Receptor tyrosine-protein kinase erbB-2 | ERBB2 |
| MOL000098 | quercetin | Receptor tyrosine-protein kinase erbB-3 | ERBB3 |
| MOL000098 | quercetin | Retinoblastoma-associated protein | RB1 |
| MOL000098 | quercetin | Runt-related transcription factor 2 | RUNX2 |
| MOL000098 | quercetin | Transcription factor E2F1 | E2F1 |
| MOL000098 | quercetin | Transforming growth factor beta-1 | TGFB1 |
| MOL000098 | quercetin | Vascular endothelial growth factor A | VEGFA |
| MOL000173 | wogonin | Fibronectin | FN1 |
| MOL000173 | wogonin | Vascular endothelial growth factor receptor 2 | KDR |
| MOL000173 | wogonin | Androgen receptor | AR |
| MOL000173 | wogonin | Apoptosis regulator BAX | BAX |
| MOL000173 | wogonin | Apoptosis regulator Bcl-2 | BCL2 |
| MOL000173 | wogonin | Caspase-3 | CASP3 |
| MOL000173 | wogonin | C-C motif chemokine 2 | CCL2 |
| MOL000173 | wogonin | Cell division protein kinase 2 | CDK2 |
| MOL000173 | wogonin | Cyclin-dependent kinase inhibitor 1 | CDKN1A |
| MOL000173 | wogonin | G1/S-specific cyclin-D1 | CCND1 |
| MOL000173 | wogonin | Glycogen synthase kinase-3 beta | GSK3B |
| MOL000173 | wogonin | Interleukin-6 | IL6 |
| MOL000173 | wogonin | Interstitial collagenase | MMP1 |
| MOL000173 | wogonin | Mitogen-activated protein kinase 14 | MAPK14 |
| MOL000173 | wogonin | Peroxisome proliferator activated receptor gamma | PPARG |
| MOL000173 | wogonin | Phosphatidylinositol-4,5-bisphosphate 3-kinase catalytic subunit, gamma isoform | PIK3CG |
| MOL000173 | wogonin | Prostaglandin E2 receptor EP3 subtype | PTGER3 |
| MOL000173 | wogonin | Prostaglandin G/H synthase 1 | PTGS1 |
| MOL000173 | wogonin | Prostaglandin G/H synthase 2 | PTGS2 |
| MOL000173 | wogonin | Protein kinase C delta type | PRKCD |
| MOL000173 | wogonin | RAC-alpha serine/threonine-protein kinase | AKT1 |
| MOL000173 | wogonin | Transcription factor AP-1 | JUN |
| MOL000173 | wogonin | Transcription factor p65 | RELA |
| MOL000173 | wogonin | Vascular endothelial growth factor receptor 2 | VEGFA |
| MOL000211 | Mairin | Progesterone receptor | PGR |
| MOL000228 | (2R)-7-hydroxy-5-methoxy-2-phenylchroman-4-one | Muscarinic acetylcholine receptor M1 | CHRM1 |
| MOL000228 | (2R)-7-hydroxy-5-methoxy-2-phenylchroman-4-one | Neuronal acetylcholine receptor protein, alpha-7 chain | CHRNA7 |
| MOL000228 | (2R)-7-hydroxy-5-methoxy-2-phenylchroman-4-one | Phosphatidylinositol-4,5-bisphosphate 3-kinase catalytic subunit, gamma isoform | PIK3CG |
| MOL000228 | (2R)-7-hydroxy-5-methoxy-2-phenylchroman-4-one | Prostaglandin G/H synthase 1 | PTGS1 |
| MOL000228 | (2R)-7-hydroxy-5-methoxy-2-phenylchroman-4-one | Prostaglandin G/H synthase 2 | PTGS2 |
| MOL000358 | beta-sitosterol | Transforming growth factor beta-1 | TGFB1 |
| MOL000358 | beta-sitosterol | Neuronal acetylcholine receptor protein, alpha-7 chain | CHRNA7 |
| MOL000358 | beta-sitosterol | Apoptosis regulator BAX | BAX |
| MOL000358 | beta-sitosterol | Apoptosis regulator Bcl-2 | BCL2 |
| MOL000358 | beta-sitosterol | Caspase-3 | CASP3 |
| MOL000358 | beta-sitosterol | Protein kinase C alpha type | PRKCA |
| MOL000358 | beta-sitosterol | Transcription factor AP-1 | JUN |
| MOL000358 | beta-sitosterol | Transforming growth factor beta-1 | TGFB1 |
| MOL000359 | sitosterol | Progesterone receptor | PGR |
| MOL000422 | kaempferol | Acetylcholinesterase | ACHE |
| MOL000422 | kaempferol | Androgen receptor | AR |
| MOL000422 | kaempferol | Apoptosis regulator BAX | BAX |
| MOL000422 | kaempferol | Apoptosis regulator Bcl-2 | BCL2 |
| MOL000422 | kaempferol | Aryl hydrocarbon receptor | AHR |
| MOL000422 | kaempferol | Cell division control protein 2 homolog | CDK1 |
| MOL000422 | kaempferol | Cytochrome P450 1A1 | CYP1A1 |
| MOL000422 | kaempferol | Cytochrome P450 3A4 | CYP3A4 |
| MOL000422 | kaempferol | Heme oxygenase 1 | HMOX1 |
| MOL000422 | kaempferol | Insulin receptor | INSR |
| MOL000422 | kaempferol | Intercellular adhesion molecule 1 | ICAM1 |
| MOL000422 | kaempferol | Interstitial collagenase | MMP1 |
| MOL000422 | kaempferol | Mitogen-activated protein kinase 8 | MAPK8 |
| MOL000422 | kaempferol | Nuclear receptor subfamily 1 group I member 2 | NR1I2 |
| MOL000422 | kaempferol | Peroxisome proliferator-activated receptor gamma | PPARG |
| MOL000422 | kaempferol | Phosphatidylinositol-4,5-bisphosphate 3-kinase catalytic subunit, gamma isoform | PIK3CG |
| MOL000422 | kaempferol | RAC-alpha serine/threonine-protein kinase | AKT1 |
| MOL000422 | kaempferol | Solute carrier family 2, facilitated glucose transporter member 4 | SLC2A4 |
| MOL000422 | kaempferol | Transcription factor AP-1 | JUN |
| MOL000422 | kaempferol | Vascular cell adhesion protein 1 | VCAM1 |
| MOL000449 | Stigmasterol | 5-hydroxytryptamine 2A receptor | HTR2A |
| MOL000449 | Stigmasterol | Muscarinic acetylcholine receptor M1 | CHRM1 |
| MOL000449 | Stigmasterol | Neuronal acetylcholine receptor protein, alpha-7 chain | CHRNA7 |
| MOL000449 | Stigmasterol | Nuclear receptor coactivator 1 | NCOA1 |
| MOL000449 | Stigmasterol | Progesterone receptor | PGR |
| MOL000449 | Stigmasterol | Prostaglandin G/H synthase 1 | PTGS1 |
| MOL000449 | Stigmasterol | Prostaglandin G/H synthase 2 | PTGS2 |
| MOL000525 | Norwogonin | Androgen receptor | AR |
| MOL000525 | Norwogonin | Cell division protein kinase 2 | CDK2 |
| MOL000525 | Norwogonin | Peroxisome proliferator activated receptor gamma | PPARG |
| MOL000525 | Norwogonin | Phosphatidylinositol-4,5-bisphosphate 3-kinase catalytic subunit, gamma isoform | PIK3CG |
| MOL000525 | Norwogonin | Prostaglandin G/H synthase 1 | PTGS1 |
| MOL000525 | Norwogonin | Prostaglandin G/H synthase 2 | PTGS2 |
| MOL000552 | 5,2'-Dihydroxy-6,7,8-trimethoxyflavone | Androgen receptor | AR |
| MOL000552 | 5,2'-Dihydroxy-6,7,8-trimethoxyflavone | DNA topoisomerase II | TOP2B |
| MOL000552 | 5,2'-Dihydroxy-6,7,8-trimethoxyflavone | Estrogen receptor beta | ESR2 |
| MOL000552 | 5,2'-Dihydroxy-6,7,8-trimethoxyflavone | Nitric-oxide synthase, endothelial | NOS3 |
| MOL000552 | 5,2'-Dihydroxy-6,7,8-trimethoxyflavone | Nuclear receptor coactivator 1 | NCOA1 |
| MOL000552 | 5,2'-Dihydroxy-6,7,8-trimethoxyflavone | Prostaglandin G/H synthase 1 | PTGS1 |
| MOL000552 | 5,2'-Dihydroxy-6,7,8-trimethoxyflavone | Prostaglandin G/H synthase 2 | PTGS2 |
| MOL000552 | 5,2'-Dihydroxy-6,7,8-trimethoxyflavone | Vascular endothelial growth factor receptor 2 | KDR |
| MOL000791 | bicuculline | Corticoliberin | CRH |
| MOL000791 | bicuculline | Gap junction alpha-1 protein | GJA1 |
| MOL000791 | bicuculline | Gonadotropin-releasing hormone receptor | GNRHR |
| MOL000791 | bicuculline | Metabotropic glutamate receptor 1 | GRM1 |
| MOL000791 | bicuculline | Metabotropic glutamate receptor 5 | GRM5 |
| MOL000791 | bicuculline | Progonadoliberin-1 | GNRH1 |
| MOL001458 | coptisine | Androgen receptor | AR |
| MOL001458 | coptisine | Nitric-oxide synthase, endothelial | NOS3 |
| MOL001458 | coptisine | Prostaglandin G/H synthase 1 | PTGS1 |
| MOL001458 | coptisine | Prostaglandin G/H synthase 2 | PTGS2 |
| MOL001494 | Mandenol | Prostaglandin G/H synthase 1 | PTGS1 |
| MOL001494 | Mandenol | Prostaglandin G/H synthase 2 | PTGS2 |
| MOL001495 | Ethyl linolenate | Prostaglandin G/H synthase 1 | PTGS1 |
| MOL001689 | acacetin | Androgen receptor | AR |
| MOL001689 | acacetin | Apoptosis regulator BAX | BAX |
| MOL001689 | acacetin | Apoptosis regulator Bcl-2 | BCL2 |
| MOL001689 | acacetin | Caspase-3 | CASP3 |
| MOL001689 | acacetin | Cell division protein kinase 2 | CDK2 |
| MOL001689 | acacetin | Cyclin-dependent kinase inhibitor 1 | CDKN1A |
| MOL001689 | acacetin | Cytochrome P450 19A1 | CYP19A1 |
| MOL001689 | acacetin | Fatty acid synthase | FASN |
| MOL001689 | acacetin | Nuclear receptor coactivator 1 | NCOA1 |
| MOL001689 | acacetin | Phosphatidylinositol-4,5-bisphosphate 3-kinase catalytic subunit, gamma isoform | PIK3CG |
| MOL001689 | acacetin | Prostaglandin G/H synthase 1 | PTGS1 |
| MOL001689 | acacetin | Prostaglandin G/H synthase 2 | PTGS2 |
| MOL001689 | acacetin | Transcription factor p65 | RELA |
| MOL001941 | Ammidin | Muscarinic acetylcholine receptor M1 | CHRM1 |
| MOL001941 | Ammidin | Phosphatidylinositol-4,5-bisphosphate 3-kinase catalytic subunit, gamma isoform | PIK3CG |
| MOL001941 | Ammidin | Prostaglandin G/H synthase 2 | PTGS2 |
| MOL001942 | isoimperatorin | Prostaglandin G/H synthase 2 | PTGS2 |
| MOL002644 | Phellopterin | Muscarinic acetylcholine receptor M1 | CHRM1 |
| MOL002644 | Phellopterin | Neuronal acetylcholine receptor protein, alpha-7 chain | CHRNA7 |
| MOL002644 | Phellopterin | Prostaglandin G/H synthase 2 | PTGS2 |
| MOL002714 | baicalein | Androgen receptor | AR |
| MOL002714 | baicalein | Apoptosis regulator BAX | BAX |
| MOL002714 | baicalein | Apoptosis regulator Bcl-2 | BCL2 |
| MOL002714 | baicalein | Aryl hydrocarbon receptor | AHR |
| MOL002714 | baicalein | Caspase-3 | CASP3 |
| MOL002714 | baicalein | Cell division control protein 2 homolog | CDK1 |
| MOL002714 | baicalein | Fos-related antigen 1 | FOSL1 |
| MOL002714 | baicalein | G2/mitotic-specific cyclin-B1 | CCNB1 |
| MOL002714 | baicalein | Hypoxia-inducible factor 1-alpha | HIF1A |
| MOL002714 | baicalein | Matrix metalloproteinase-9 | MMP9 |
| MOL002714 | baicalein | Nuclear receptor coactivator 1 | NCOA1 |
| MOL002714 | baicalein | Phosphatidylinositol-4,5-bisphosphate 3-kinase catalytic subunit, gamma isoform | PIK3CG |
| MOL002714 | baicalein | Prostaglandin G/H synthase 1 | PTGS1 |
| MOL002714 | baicalein | Prostaglandin G/H synthase 2 | PTGS2 |
| MOL002714 | baicalein | Proto-oncogene c-Fos | FOS |
| MOL002714 | baicalein | RAC-alpha serine/threonine-protein kinase | AKT1 |
| MOL002714 | baicalein | Transcription factor p65 | RELA |
| MOL002714 | baicalein | Vascular endothelial growth factor A | VEGFA |
| MOL002773 | beta-carotene | Catenin beta-1 | CTNNB1 |
| MOL002773 | beta-carotene | Caveolin-1 | CAV1 |
| MOL002773 | beta-carotene | Cytochrome P450 3A4 | CYP3A4 |
| MOL002773 | beta-carotene | Gap junction alpha-1 protein | GJA1 |
| MOL002773 | beta-carotene | Myc proto-oncogene protein | MYC |
| MOL002773 | beta-carotene | RAC-alpha serine/threonine-protein kinase | AKT1 |
| MOL002773 | beta-carotene | Serum albumin | ALB |
| MOL002881 | Diosmetin | Nuclear receptor coactivator 1 | NCOA1 |
| MOL002881 | Diosmetin | Prostaglandin G/H synthase 1 | PTGS1 |
| MOL002881 | Diosmetin | Prostaglandin G/H synthase 2 | PTGS2 |
| MOL002897 | epiberberine | Androgen receptor | AR |
| MOL002897 | epiberberine | Nitric-oxide synthase, endothelial | NOS3 |
| MOL002897 | epiberberine | Prostaglandin G/H synthase 2 | PTGS2 |
| MOL002909 | 5,7,2,5-tetrahydroxy-8,6-dimethoxyflavone | Androgen receptor | AR |
| MOL002909 | 5,7,2,5-tetrahydroxy-8,6-dimethoxyflavone | DNA topoisomerase II | TOP2B |
| MOL002909 | 5,7,2,5-tetrahydroxy-8,6-dimethoxyflavone | Prostaglandin G/H synthase 2 | PTGS2 |
| MOL002910 | Carthamidin | Prostaglandin G/H synthase 1 | PTGS1 |
| MOL002910 | Carthamidin | Prostaglandin G/H synthase 2 | PTGS2 |
| MOL002913 | Dihydrobaicalin_qt | Prostaglandin G/H synthase 1 | PTGS1 |
| MOL002913 | Dihydrobaicalin_qt | Prostaglandin G/H synthase 2 | PTGS2 |
| MOL002915 | Salvigenin | Acetylcholinesterase | ACHE |
| MOL002915 | Salvigenin | Nitric-oxide synthase, endothelial | NOS3 |
| MOL002915 | Salvigenin | Prostaglandin G/H synthase 1 | PTGS1 |
| MOL002915 | Salvigenin | Prostaglandin G/H synthase 2 | PTGS2 |
| MOL002917 | 5,2',6'-Trihydroxy-7,8-dimethoxyflavone | Androgen receptor | AR |
| MOL002917 | 5,2',6'-Trihydroxy-7,8-dimethoxyflavone | Cell division protein kinase 2 | CDK2 |
| MOL002917 | 5,2',6'-Trihydroxy-7,8-dimethoxyflavone | DNA topoisomerase II | TOP2B |
| MOL002917 | 5,2',6'-Trihydroxy-7,8-dimethoxyflavone | Estrogen receptor beta | ESR2 |
| MOL002917 | 5,2',6'-Trihydroxy-7,8-dimethoxyflavone | Phosphatidylinositol-4,5-bisphosphate 3-kinase catalytic subunit, gamma isoform | PIK3CG |
| MOL002917 | 5,2',6'-Trihydroxy-7,8-dimethoxyflavone | Prostaglandin G/H synthase 1 | PTGS1 |
| MOL002917 | 5,2',6'-Trihydroxy-7,8-dimethoxyflavone | Prostaglandin G/H synthase 2 | PTGS2 |
| MOL002925 | 5,7,2',6'-Tetrahydroxyflavone | Androgen receptor | AR |
| MOL002925 | 5,7,2',6'-Tetrahydroxyflavone | Prostaglandin G/H synthase 1 | PTGS1 |
| MOL002925 | 5,7,2',6'-Tetrahydroxyflavone | Prostaglandin G/H synthase 2 | PTGS2 |
| MOL002926 | dihydrooroxylin A | Cell division control protein 2 homolog | CDK1 |
| MOL002926 | dihydrooroxylin A | Cell division protein kinase 5 | CDK5 |
| MOL002926 | dihydrooroxylin A | Estrogen receptor beta | ESR2 |
| MOL002926 | dihydrooroxylin A | Glycogen synthase kinase-3 beta | GSK3B |
| MOL002926 | dihydrooroxylin A | Mitogen-activated protein kinase 14 | MAPK14 |
| MOL002927 | Skullcapflavone II | Androgen receptor | AR |
| MOL002927 | Skullcapflavone II | DNA topoisomerase II | TOP2B |
| MOL002927 | Skullcapflavone II | Nitric-oxide synthase, endothelial | NOS3 |
| MOL002927 | Skullcapflavone II | Nuclear receptor coactivator 1 | NCOA1 |
| MOL002927 | Skullcapflavone II | Prostaglandin G/H synthase 1 | PTGS1 |
| MOL002927 | Skullcapflavone II | Prostaglandin G/H synthase 2 | PTGS2 |
| MOL002927 | Skullcapflavone II | Vascular endothelial growth factor receptor 2 | KDR |
| MOL002928 | oroxylin a | Androgen receptor | AR |
| MOL002928 | oroxylin a | Apoptosis regulator Bcl-2 | BCL2 |
| MOL002928 | oroxylin a | Caspase-3 | CASP3 |
| MOL002928 | oroxylin a | Cell division control protein 2 homolog | CDK1 |
| MOL002928 | oroxylin a | G2/mitotic-specific cyclin-B1 | CCNB1 |
| MOL002928 | oroxylin a | Interleukin-6 | IL6 |
| MOL002928 | oroxylin a | Nuclear receptor coactivator 1 | NCOA1 |
| MOL002928 | oroxylin a | Phosphatidylinositol-4,5-bisphosphate 3-kinase catalytic subunit, gamma isoform | PIK3CG |
| MOL002928 | oroxylin a | Prostaglandin G/H synthase 1 | PTGS1 |
| MOL002928 | oroxylin a | Prostaglandin G/H synthase 2 | PTGS2 |
| MOL002932 | Panicolin | Androgen receptor | AR |
| MOL002932 | Panicolin | Cell division protein kinase 2 | CDK2 |
| MOL002932 | Panicolin | Estrogen receptor beta | ESR2 |
| MOL002932 | Panicolin | Nuclear receptor coactivator 1 | NCOA1 |
| MOL002932 | Panicolin | Phosphatidylinositol-4,5-bisphosphate 3-kinase catalytic subunit, gamma isoform | PIK3CG |
| MOL002932 | Panicolin | Prostaglandin G/H synthase 1 | PTGS1 |
| MOL002932 | Panicolin | Prostaglandin G/H synthase 2 | PTGS2 |
| MOL002933 | 5,7,4'-Trihydroxy-8-methoxyflavone | Androgen receptor | AR |
| MOL002933 | 5,7,4'-Trihydroxy-8-methoxyflavone | Cell division protein kinase 2 | CDK2 |
| MOL002933 | 5,7,4'-Trihydroxy-8-methoxyflavone | Glycogen synthase kinase-3 beta | GSK3B |
| MOL002933 | 5,7,4'-Trihydroxy-8-methoxyflavone | Mitogen-activated protein kinase 14 | MAPK14 |
| MOL002933 | 5,7,4'-Trihydroxy-8-methoxyflavone | Peroxisome proliferator activated receptor gamma | PPARG |
| MOL002933 | 5,7,4'-Trihydroxy-8-methoxyflavone | Phosphatidylinositol-4,5-bisphosphate 3-kinase catalytic subunit, gamma isoform | PIK3CG |
| MOL002933 | 5,7,4'-Trihydroxy-8-methoxyflavone | Prostaglandin G/H synthase 1 | PTGS1 |
| MOL002933 | 5,7,4'-Trihydroxy-8-methoxyflavone | Prostaglandin G/H synthase 2 | PTGS2 |
| MOL002934 | NEOBAICALEIN | Androgen receptor | AR |
| MOL002934 | NEOBAICALEIN | DNA topoisomerase II | TOP2B |
| MOL002934 | NEOBAICALEIN | Estrogen receptor beta | ESR2 |
| MOL002934 | NEOBAICALEIN | Glycogen synthase kinase-3 beta | GSK3B |
| MOL002934 | NEOBAICALEIN | Peroxisome proliferator activated receptor gamma | PPARG |
| MOL002934 | NEOBAICALEIN | Prostaglandin G/H synthase 2 | PTGS2 |
| MOL002937 | DIHYDROOROXYLIN | Nuclear receptor coactivator 1 | NCOA1 |
| MOL002937 | DIHYDROOROXYLIN | Prostaglandin G/H synthase 1 | PTGS1 |
| MOL002937 | DIHYDROOROXYLIN | Prostaglandin G/H synthase 2 | PTGS2 |
| MOL003014 | secologanic dibutylacetal_qt | Prostaglandin G/H synthase 2 | PTGS2 |
| MOL003117 | Ioniceracetalides B_qt | Prostaglandin G/H synthase 2 | PTGS2 |
| MOL003283 | (2R,3R,4S)-4-(4-hydroxy-3-methoxy-phenyl)-7-methoxy-2,3-dimethylol-tetralin-6-ol | Estrogen receptor beta | ESR2 |
| MOL003290 | (3R,4R)-3,4-bis[(3,4-dimethoxyphenyl)methyl]oxolan-2-one | Prostaglandin G/H synthase 2 | PTGS2 |
| MOL003306 | ACon1_001697 | Nuclear receptor coactivator 1 | NCOA1 |
| MOL003306 | ACon1_001697 | Prostaglandin G/H synthase 1 | PTGS1 |
| MOL003306 | ACon1_001697 | Prostaglandin G/H synthase 2 | PTGS2 |
| MOL003308 | (+)-pinoresinol monomethyl ether-4-D-beta-glucoside_qt | Nuclear receptor coactivator 1 | NCOA1 |
| MOL003308 | (+)-pinoresinol monomethyl ether-4-D-beta-glucoside_qt | Prostaglandin G/H synthase 2 | PTGS2 |
| MOL003322 | FORSYTHINOL | Nuclear receptor coactivator 1 | NCOA1 |
| MOL003322 | FORSYTHINOL | Prostaglandin G/H synthase 2 | PTGS2 |
| MOL003365 | Lactucasterol | Estrogen receptor beta | ESR2 |
| MOL003365 | Lactucasterol | Nuclear receptor coactivator 1 | NCOA1 |
| MOL003370 | Onjixanthone I | Estrogen receptor beta | ESR2 |
| MOL003370 | Onjixanthone I | Phosphatidylinositol-4,5-bisphosphate 3-kinase catalytic subunit, gamma isoform | PIK3CG |
| MOL003370 | Onjixanthone I | Prostaglandin G/H synthase 1 | PTGS1 |
| MOL003370 | Onjixanthone I | Prostaglandin G/H synthase 2 | PTGS2 |
| MOL003851 | Isoramanone | Nuclear receptor coactivator 1 | NCOA1 |
| MOL003851 | Isoramanone | Progesterone receptor | PGR |
| MOL003975 | icosa-11,14,17-trienoic acid methyl ester | Hepatocyte nuclear factor 4-alpha | HNF4A |
| MOL003975 | icosa-11,14,17-trienoic acid methyl ester | Insulin | INS |
| MOL003975 | icosa-11,14,17-trienoic acid methyl ester | Nuclear receptor coactivator 1 | NCOA1 |
| MOL003975 | icosa-11,14,17-trienoic acid methyl ester | Toll-like receptor 4 | TLR4 |
| MOL004350 | Ruvoside_qt | Nuclear receptor coactivator 1 | NCOA1 |
| MOL004355 | Spinasterol | Progesterone receptor | PGR |
| MOL004653 | (+)-Anomalin | DNA topoisomerase II | TOP2B |
| MOL004653 | (+)-Anomalin | Prostaglandin G/H synthase 2 | PTGS2 |
| MOL005100 | 5,7-dihydroxy-2-(3-hydroxy-4-methoxyphenyl)chroman-4-one | DNA topoisomerase II | TOP2B |
| MOL005100 | 5,7-dihydroxy-2-(3-hydroxy-4-methoxyphenyl)chroman-4-one | Nuclear receptor coactivator 1 | NCOA1 |
| MOL005100 | 5,7-dihydroxy-2-(3-hydroxy-4-methoxyphenyl)chroman-4-one | Phosphatidylinositol-4,5-bisphosphate 3-kinase catalytic subunit, gamma isoform | PIK3CG |
| MOL005100 | 5,7-dihydroxy-2-(3-hydroxy-4-methoxyphenyl)chroman-4-one | Prostaglandin G/H synthase 1 | PTGS1 |
| MOL005100 | 5,7-dihydroxy-2-(3-hydroxy-4-methoxyphenyl)chroman-4-one | Prostaglandin G/H synthase 2 | PTGS2 |
| MOL005530 | Hydroxygenkwanin | Phosphatidylinositol-4,5-bisphosphate 3-kinase catalytic subunit, gamma isoform | PIK3CG |
| MOL005530 | Hydroxygenkwanin | Prostaglandin G/H synthase 1 | PTGS1 |
| MOL005530 | Hydroxygenkwanin | Prostaglandin G/H synthase 2 | PTGS2 |
| MOL006756 | Schottenol | Progesterone receptor | PGR |
| MOL007154 | tanshinone iia | Myc proto-oncogene protein | MYC |
| MOL007154 | tanshinone iia | Nucleophosmin | NPM1 |
| MOL007165 | 10α-cucurbita-5,24-diene-3β-ol | Progesterone receptor | PGR |
| MOL007179 | Linolenic acid ethyl ester | Prostaglandin G/H synthase 1 | PTGS1 |
| MOL007179 | Linolenic acid ethyl ester | Prostaglandin G/H synthase 2 | PTGS2 |
| MOL008206 | Moslosooflavone | Androgen receptor | AR |
| MOL008206 | Moslosooflavone | Cell division protein kinase 2 | CDK2 |
| MOL008206 | Moslosooflavone | Estrogen receptor beta | ESR2 |
| MOL008206 | Moslosooflavone | Glycogen synthase kinase-3 beta | GSK3B |
| MOL008206 | Moslosooflavone | Mitogen-activated protein kinase 14 | MAPK14 |
| MOL008206 | Moslosooflavone | Neuronal acetylcholine receptor protein, alpha-7 chain | CHRNA7 |
| MOL008206 | Moslosooflavone | Nuclear receptor coactivator 1 | NCOA1 |
| MOL008206 | Moslosooflavone | Peroxisome proliferator activated receptor gamma | PPARG |
| MOL008206 | Moslosooflavone | Phosphatidylinositol-4,5-bisphosphate 3-kinase catalytic subunit, gamma isoform | PIK3CG |
| MOL008206 | Moslosooflavone | Prostaglandin G/H synthase 1 | PTGS1 |
| MOL008206 | Moslosooflavone | Prostaglandin G/H synthase 2 | PTGS2 |
| MOL012245 | 5,7,4'-trihydroxy-6-methoxyflavanone | Prostaglandin G/H synthase 1 | PTGS1 |
| MOL012245 | 5,7,4'-trihydroxy-6-methoxyflavanone | Prostaglandin G/H synthase 2 | PTGS2 |
| MOL012246 | 5,7,4'-trihydroxy-8-methoxyflavanone | Phosphatidylinositol-4,5-bisphosphate 3-kinase catalytic subunit, gamma isoform | PIK3CG |
| MOL012246 | 5,7,4'-trihydroxy-8-methoxyflavanone | Prostaglandin G/H synthase 1 | PTGS1 |
| MOL012246 | 5,7,4'-trihydroxy-8-methoxyflavanone | Prostaglandin G/H synthase 2 | PTGS2 |
| MOL012266 | rivularin | Androgen receptor | AR |
| MOL012266 | rivularin | DNA topoisomerase II | TOP2B |
| MOL012266 | rivularin | Estrogen receptor beta | ESR2 |
| MOL012266 | rivularin | Nitric-oxide synthase, endothelial | NOS3 |
| MOL012266 | rivularin | Nuclear receptor coactivator 1 | NCOA1 |
| MOL012266 | rivularin | Prostaglandin G/H synthase 1 | PTGS1 |
| MOL012266 | rivularin | Prostaglandin G/H synthase 2 | PTGS2 |
| MOL012266 | rivularin | Vascular endothelial growth factor receptor 2 | KDR |
| MOL013076 | (8S,9R)-9-hydroxy-8-(2-hydroxypropan-2-yl)-8,9-dihydrofuro[2,3-h]chromen-2-one | Androgen receptor | AR |
| MOL013078 | praeruptorin E | DNA topoisomerase II | TOP2B |
| MOL013078 | praeruptorin E | Nuclear receptor coactivator 1 | NCOA1 |
| MOL013079 | dl-praeruptorin a | Transcription factor p65 | RELA |
| MOL013087 | Peucedanocoumarin II | DNA topoisomerase II | TOP2B |
| MOL013087 | Peucedanocoumarin II | Nuclear receptor coactivator 1 | NCOA1 |
| MOL013087 | Peucedanocoumarin II | Prostaglandin G/H synthase 2 | PTGS2 |
| MOL013098 | [(9R)-8,8-dimethyl-2-oxo-9,10-dihydropyrano[6,5-h]chromen-9-yl] (Z)-2-methylbut-2-enoate | Prostaglandin G/H synthase 1 | PTGS1 |
| MOL013098 | [(9R)-8,8-dimethyl-2-oxo-9,10-dihydropyrano[6,5-h]chromen-9-yl] (Z)-2-methylbut-2-enoate | Prostaglandin G/H synthase 2 | PTGS2 |
| MOL013101 | rutarin_qt | Acetylcholinesterase | ACHE |
| MOL013101 | rutarin_qt | Kappa-type opioid receptor | OPRK1 |
| MOL013101 | rutarin_qt | Neuronal acetylcholine receptor subunit alpha-4 | CHRNA4 |
| MOL013101 | rutarin_qt | Neuronal acetylcholine receptor subunit alpha-7 | CHRNA7 |
| MOL013101 | rutarin_qt | Prostaglandin G/H synthase 2 | PTGS2 |
| MOL013101 | rutarin_qt | Prothrombin | F2 |
| MOL013103 | 532-16-1 | Prostaglandin G/H synthase 2 | PTGS2 |
